# Recent Advances of Modified Ni (Co, Fe)-Based LDH 2D Materials for Water Splitting

**DOI:** 10.3390/molecules28031475

**Published:** 2023-02-03

**Authors:** Chenguang Li, Yupeng Bao, Enzhou Liu, Binran Zhao, Tao Sun

**Affiliations:** School of Chemical Engineering, Xi’an Key Laboratory of Special Energy Materials, Northwest University, Xi’an 710069, China

**Keywords:** water splitting, LDH materials, hydrogen evolution reaction, oxygen evolution reaction, electrocatalytic performance

## Abstract

Water splitting technology is an efficient approach to produce hydrogen (H_2_) as an energy carrier, which can address the problems of environmental deterioration and energy shortage well, as well as establishment of a clean and sustainable hydrogen economy powered by renewable energy sources due to the green reaction of H_2_ with O_2_. The efficiency of H_2_ production by water splitting technology is intimately related with the reactions on the electrode. Nowadays, the efficient electrocatalysts in water splitting reactions are the precious metal-based materials, i.e., Pt/C, RuO_2_, and IrO_2_. Ni (Co, Fe)-based layered double hydroxides (LDH) two-dimensional (2D) materials are the typical non-precious metal-based materials in water splitting with their advantages including low cost, excellent electrocatalytic performance, and simple preparation methods. They exhibit great potential for the substitution of precious metal-based materials. This review summarizes the recent progress of Ni (Co, Fe)-based LDH 2D materials for water splitting, and mainly focuses on discussing and analyzing the different strategies for modifying LDH materials towards high electrocatalytic performance. We also discuss recent achievements, including their electronic structure, electrocatalytic performance, catalytic center, preparation process, and catalytic mechanism. Furthermore, the characterization progress in revealing the electronic structure and catalytic mechanism of LDH is highlighted in this review. Finally, we put forward some future perspectives relating to design and explore advanced LDH catalysts in water splitting.

## 1. Introduction

Water splitting technology to produce hydrogen (H_2_) as an energy carrier is always regarded as an efficient approach to address the problems of environmental deterioration and energy shortage. It can establish a clean and sustainable hydrogen economy powered by renewable energy sources due to the high-energy capacity of H_2_ and the green reaction of H_2_ with O_2_ [1,2,3]. This technology contains two important reactions in the electrolytic cell. The first one is the hydrogen evolution reaction (HER, 2H^+^ + 2e^−^ → H_2_) on the cathode, and second one is the oxygen evolution reaction (OER, 2H_2_O → 4H^+^ + 4e^−^ + O_2_) on the anode [3,4]. These two reactions are all that is needed for the catalysts to accelerate. Nowadays, the most efficiently active HER and OER electrocatalysts are precious metal-based materials, e.g., Pt/C, RuO_2_, and IrO_2_, which possess the high exchange current density (*j_0_*), small Tafel slope, and low overpotential [3,5]. However, their prohibitive cost and scarcity greatly hinder the large-scale application of water splitting. Therefore, developing non-precious metal-based HER and OER electrocatalysts are of significance and important for the large-scale application of water splitting technology for H_2_ generation.

The HER rate is dependent on H* adsorption conditions over the surface of catalysts, which closely relates with the electronic structure of the catalysts [6,7]. In general, an advanced HER electrocatalyst should possess a suitable H* adsorption energy which is neither too strong nor too weak, i.e., ΔG_H*_, as reflected by the commercial Pt/C catalyst with |ΔG_H*_| being near to zero. OER involves a four-electron transfer process, which means that the transfer of 4e^−^ and 4H^+^ from two water (H_2_O) molecules can generate one oxygen molecule. Due to this point, OER generally possesses the larger overpotential than HER because of its lower sluggish reaction kinetics, which directly determines the efficiency of electrolyser technologies and is also regarded as the bottleneck reaction of water splitting technology [8,9]. Similar to HER, the precious metal-based oxides, i.e., RuO_2_ and IrO_2_, have also been regarded as the benchmarks of OER catalysts, which possess excellent OER activity in a wide range of pH values. However, OER over RuO_2_/IrO_2_ involves a lattice oxygen insertion and removal process (i.e., the insertion/removal of O in and out of lattice, renamed as the lattice oxygen evolution reaction (LOER)), which results in a collapse of the crystal structure and leaching of Ru/Ir species, thus losing the OER activity [10]. This important problem also restricts the large-scale application of water splitting to generate H_2_ energy. Based on these analyses, the design of non-precious metal-based compounds is of great significance to replace the commercial precious metals and reduce the cost of water splitting for H_2_ generation.

**Figure 1:**
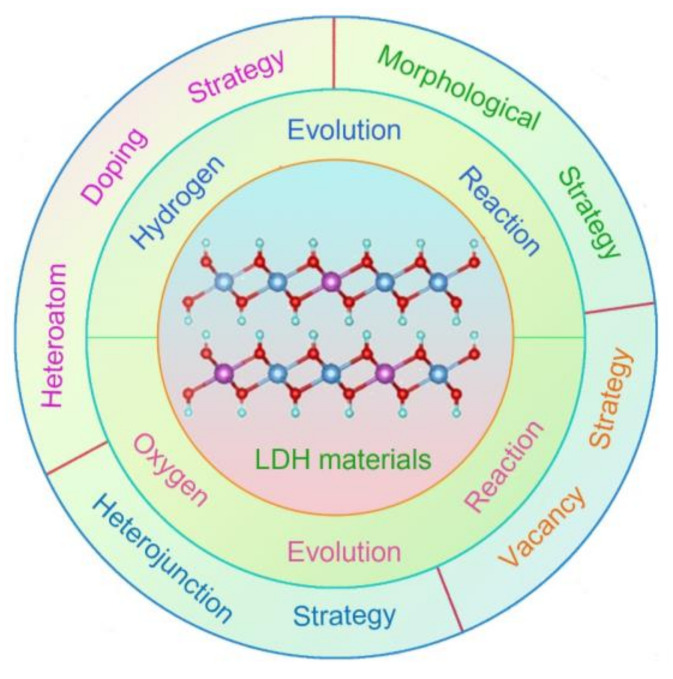
Modification strategies of Ni (Co, Fe)-based LDH materials as water splitting electrocatalysts.

Nowadays, a large number of non-precious metal-based materials have been widely developed in water splitting technology, including carbon materials, metals, metal oxides/hydroxides, sulfides, and phosphides [3,9,11,12,13,14,15,16,17]. Among these non-precious metal-based materials, metal-based layered double hydroxides (LDH) exhibit the great promising potential for the substitution of precious metal-based catalysts due to their low cost, simple preparation methods, and excellent electrocatalytic performance in water splitting, as well as the easier tunable chemistry in regulating electrocatalytic activity [12,18]. LDHs is a layered hydrotalcite-like compound composed of octahedral MO_6_ stacked layers sharing the edge. Among them, [M^2+^_1−x_M^3+^_x_(OH)_2_]^x+^ (M usually refers to the transition metal in the first row) represents positively charged metal hydroxide layers, and [A_x/n_•H_2_O]^n−^ represents water molecules and anions (CO_3_^2−^, Cl^−^, NO_3_^−^, VO_4_^−^, etc.) for balancing positive charges between layers. LDH materials (such as NiFe-, NiCo-, CoFe-hydroxides, etc.) have become one of the most promising abundant earth-transition metals-based electrocatalysts because of its adjustable and changeable interlayer anions. For metal sulfides and phosphides, their structures are not stable and easily reconstruct into metal hydroxides due to their sensitivity to O species during water splitting reactions, especially for the OER [11,19]. Then, the newly formed metal hydroxides can efficiently catalyze OER and HER. Similar, non-precious metals are also sensitive to O species and are more easily oxidized [20,21], then form metal hydroxides in OER and HER. In most studies, metal hydroxides are inevitable during OER and HER, which are also widely regarded as highly active catalytic sites with superior activity of current density. Ni (Co, Fe)-based LDH materials featured by 2D morphology are the most typical hydroxides as HER and OER catalysts with high performance, rich sources in the earth, and convenient preparation methods, which have grown into a category of non-precious metal-based electrocatalysts for substituting precious metal materials [12,22,23]. Nowadays, with the advantages of a high current density (reaching up to 1000 mA cm^−2^) and superior stability (1000 h), the Ni (Co, Fe)-based LDH materials exhibit a promising potential for the substitution of commercial precious metal-based materials, and also have achieved more attention in water splitting technology. As is well known, an excellent electrocatalyst should possess a high conductivity which endows the fast electron transfer from the internal catalyst to its surface to participate in the reaction with reactants. The Ni (Co, Fe)-based LDH materials have a low conductivity which greatly hinders their application in electrocatalysis. In the last several decades, a large number of Ni (Co, Fe)-based LDH materials have been constructed as electrocatalysts in water splitting [24]. Therefore, a comprehensive and systematical review focusing on summarizing these achievements on Ni (Co, Fe)-based LDH 2D materials is very important for moving forward the development of non-precious metal-based materials. This review summarizes the recent progress of Ni (Co, Fe)-based LDH materials for water splitting, which mainly focuses on discussing and analyzing the different strategies to modify LDH materials towards high electrocatalytic performance (Figure 1). This is highlighted in the area of water electrolysis, where Ni (Co, Fe)-based LDH have exhibited great promise to achieve high OER and HER activities through forming highly-intrinsic catalytic sites via different strategies. We also discuss recent achievements, including electronic structure, electrocatalytic performance, catalytic center, and preparation process. Furthermore, the characterization progress in revealing the electronic structure and catalytic mechanism of LDH is also summarized in detail. Finally, we put forward some future perspectives related to design and explore advanced LDH catalysts in water splitting.

## 2. Heteroatom Doping Strategy

Heteroatoms incorporated into the crystal structure of Ni (Co, Fe)-based LDH materials can induce the electron distribution of host atoms due to the new formed chemical bonds between heteroatoms and host atoms, which leads to the generation of new highly active catalytic sites for water splitting. Therefore, doping with the impurity atom in metallic hydroxides has been widely regarded as an effective strategy to construct a high electrocatalytic performance of LDH-based electrocatalysts. Doping with heteroatoms can easily produce a large number of oxygen vacancies (*V*_O_) due to the broken crystal structure, which can increase the conductivity of LDH as presented electronic states near the Fermi level and also make more active sites be exposed in the electrolyte, thus leading to easy electron transfer during reactions and more ratios of catalytic sites reacting with intermediates. Simultaneously, the generated *V*_O_ and heteroatoms jointly regulate the electronic states of catalytic sites, thus optimizing the adsorption energies of intermediates. In this section, we will summarize the achieved recent progress of the doping strategy in promoting the electrocatalytic performance of LDH materials, including their preparation methods, characterizations, catalytic mechanism, and theoretical studies.

### 2.1. Precious Metal Doping

**Figure 2:**
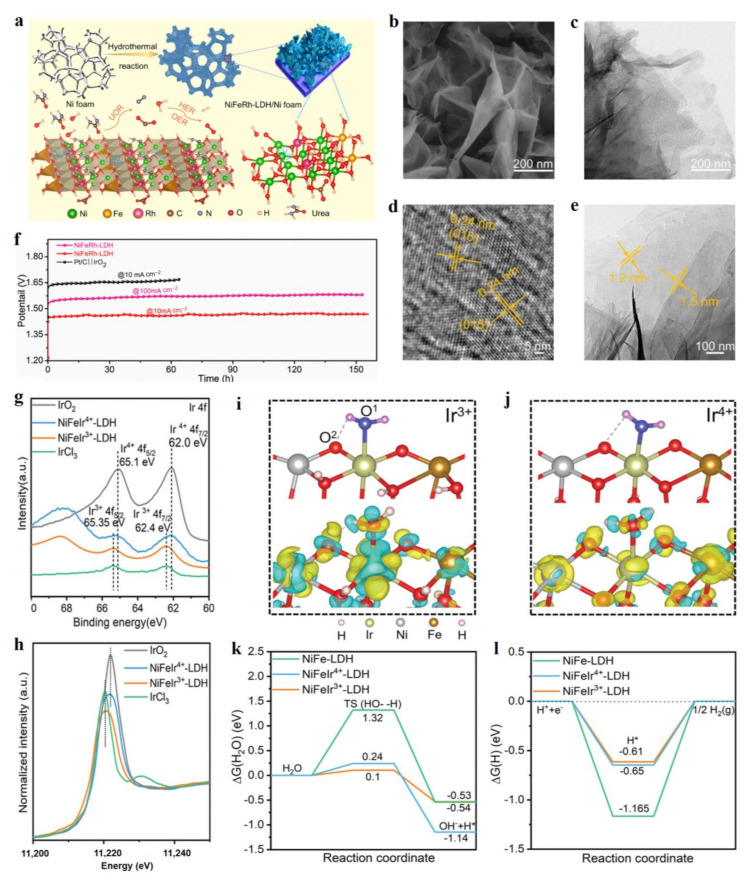
Characterizations and theoretical studies of precious metal doped LDH. (**a**) Schematic illustration of the preparation process of Rh-NiFe-LDH on nickel foam. (**b**) Scanning electron microscope (SEM) image of Rh-NiFe-LDH. (**c**–**e**) Transmission electron microscope (TEM) image of Rh-NiFe-LDH. (**f**) Durability tests of Rh-NiFe-LDH as bifunctional catalysts in water splitting cells at different current densities. (**g**) XPS spectra of Ir. (**h**) Ir L-edge XANES spectra of serial materials. (**i**,**j**) Atomic model (top) and differential-charge density (down) for H_2_O molecule adsorbed on Ir^3+^-NiFe-LDH (**i**) and Ir^4+^-NiFe-LDH. Note: yellow and blue regions represent excess and depletion of charge density, respectively. (**k**,**l**) Calculated adsorption free energy diagrams for Volmer step (**k**) and Tafel step (**l**) on serial catalysts. (**a**–**f**) Reproduced with permission [30]. Copyright 2021, Elsevier. (**g**–**l**) Reproduced with permission [32]. Copyright 2021, Wiley-VCH.

Precious metals, such as Pt, Ru, and Ir, are important in compositions to form highly-intrinsic catalytic sites for OER and HER, which possess optimized intermediate adsorption energies during reactions, thus exhibiting low overpotentials [25,26].Precious metal doping LDH can construct highly-catalytic sites for HER and OER, which develops a large number of excellent LDH catalysts in water splitting [27,28,29,30,31,32], such as Ag, Ru, Rh, Ir, and Pt. Wang et al. prepared Ni foam supporting Rh doped NiFe-LDH material by the hydrothermal method, which exhibited bifunctional electrocatalytic activities for HER and OER, as well as the urea oxidation reaction (UOR) [30]. The Rh-NiFe-LDH material, featured with thin layers of 1.2–1.5 nm (Figure 2a–e), exhibited an excellent HER and OER performance, including a low overpotential of 24 mV at 10 mA cm^−2^ for HER and 204 mV at 10 mA cm^−2^ for OER. This Rh-NiFe-LDH catalyst also showed excellent stability in a water splitting cell in the continuous 155 h test (Figure 2f). In addition, this hydrothermal method was also well applied in constructing Ir-NiFe-LDH using ethylene glycol as solvent. Fan and co-workers reported that an atomic Ir doped NiCo-LDH showed better HER and OER properties than those of commercial Pt/C and Ir/C electrodes, respectively [31]. In a water splitting cell, Ir-NiCo-LDH achieved the current density of 10 mA cm^−2^ at the ultra-low voltage of 1.45 V, showing excellent water-splitting performance, which was superior to many recent state-of-the-art bifunctional water-splitting catalysts. A single Ir atom incorporated into NiCo-LDH could generate a large number of *V*o vacancies which tuned the electron states, thus forming the highly active sites for HER and OER activities to lower their overpotentials.

The valence state of a single precious metal can efficiently tune the electrocatalytic performance of LDH materials. Wang et al. synthesized NiFe-LDH doped with Ir ions with different valence states by a simple hydrothermal method [32]. X-ray photoelectron spectra (XPS) and Ir L-edge X-ray absorption near the edge structure (XANES) spectra showed two categories of Ir-NiFe-LDH materials with Ir^3+^ and Ir^4+^ species by choosing the different Ir precursors (Figure 2g,h). The low-valence Ir^3+^-NiFe-LDH exhibited superior HER and OER activities to Ir^4+^-NiFe-LDH material, including the low overpotentials of HER (312 mV at 500 mA cm^−2^) and OER (207 mV at 10 mA cm^−2^). The density functional theory (DFT) calculation revealed that the adsorption sites changed from Ni sites to Ir^3+^/Ir^4+^ sites after the Ir ions were doped into NiFe-LDH (Figure 2i,j). On the one hand, compared with NiFe-LDH and Ir^4+^-NiFe-LDH, Ir^3+^-NiFe-LDH had the lowest adsorption energy for water molecules, thus promoting the adsorption of water molecules and accelerating the Volmer reaction of HER (Figure 2k). On the other hand, Ir^3+^ could provide more electrons to react with H_2_O molecules, thus weakening the O-H bond in water molecules and promoting the dissociation of water molecules, which facilitated the kinetics of the OER process (Figure 2i,j). In addition, the incorporation of Ir^3+^/Ir^4+^ into NiFe-LDH obviously reduced the water-dissociation energy barriers and the energy barrier for the desorption of H* species (Figure 2k,l). This work demonstrates well that the valence state of heteroatoms plays a critical role in tuning the electrocatalytic performance of LDH, and also provides an efficient approach to construct advanced LDH material through regulating the electron states of precious metals. At present, the rational design of advanced HER electrocatalysts should consider the two points of the dissociation of water molecules and the H* adsorption energy. Nowadays, the precious metal doping strategy has been widely applied in constructing excellent electrocatalysts in water splitting [33,34,35], which can form the optimized catalyst sites with the best free energy profile, thus leading to an ultrahigh mass activity based on precious metals.

### 2.2. Non-Precious Metal Doping

A large number of non-precious metal doping LDH also can construct high-performance electrocatalysts in water splitting, including Fe, V, Cr, W, Mo, and Ce [36,37]. These kinds of LDH materials usually exhibit bifunctional electrocatalytic performances in OER and HER activities. Li et al. reported a V doped NiFe-LDH material by H_2_ plasma reduction treatment and cation doping methods, which showed high HER and OER performances due to the synergistic effect of oxygen and metal vacancies, as well as V dopant [38]. The V dopant played a regulator to activate its neighboring Ni atoms in NiFe-LDH, thus leading to the optimized energy profiles of HER and OER. Vacancy defect engineering is widely regarded as the promising strategy to activate the intrinsic electrocatalytic activity of electrocatalysts, which can regulate the electronic structure of materials, thus leading to formed optimal free energy profiles [39,40]. Zhou and co-workers reported a general method for the preparation of Fe doped Ni(OH)_2_ and Co(OH)_2_ nanosheets with abundant active sites using a simple cation-exchange process, which performed extraordinary OER [41]. The enhanced OER activity of Ni_0.83_Fe_0.17_(OH)_2_ on NiFe-LDH was attributed to the defect-rich hole basal plane of Ni_0.83_Fe_0.17_(OH)_2_ generated by the cation exchange process. Firstly, the nanoporous structure and abundant Ni and O vacancies formed during the chemical etching process in the containing Fe^3+^ solution, which easily generated more exposed active sites. The O vacancies could effectively tune the valence states of Ni and Fe, thus regulating electrocatalytic activity. Secondly, Ni_0.83_Fe_0.17_(OH)_2_ exhibited better wettability than NiFe-LDH and original Ni(OH)_2_, which resulted in easier electrolyte permeation and the acceleration of the migration of hydroxyl groups and the release of oxygen. Due to the above points, Ni_0.83_Fe_0.17_(OH)_2_ exhibits a high OER performance with a low overpotential of 245 mV at 10 mA cm^−2^.

**Figure 3:**
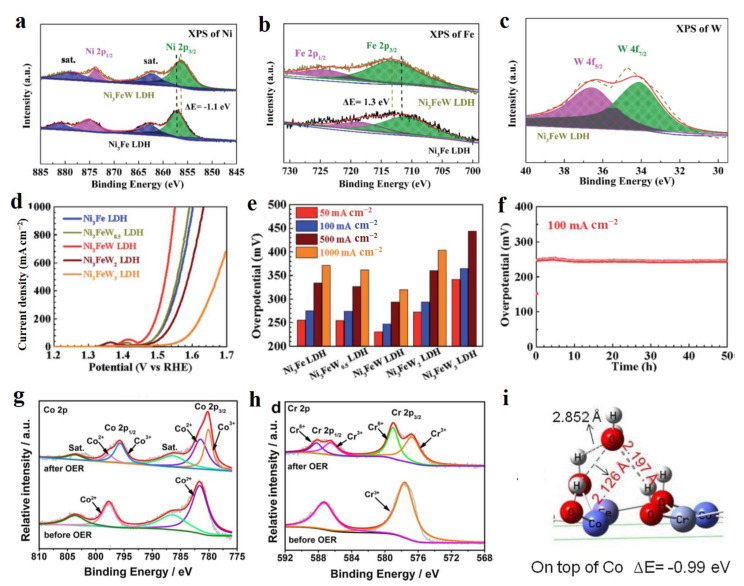
Chemical state characterizations, OER performance, and theoretical studies non-precious metal doped LDH materials. (**a**) Ni 2p XPS of W-Ni_3_Fe-LDH and Ni_3_Fe-LDH. (**b**) Fe 2p XPS of W-Ni_3_Fe-LDH and Ni_3_Fe-LDH. (**c**) W 4f XPS of W-Ni_3_Fe-LDH. (**d**) Linear sweep voltammetry (LSV) curves of serial Ni_3_Fe-LDH with different W contents. (**e**) Comparison of OER performance of serial materials at different current densities. (**f**) Long-time stability of W-Ni_3_Fe-LDH at 100 mA cm^−2^. (**g**) Co 2p XPS of Cr-CoF-LDH before and after OER. (**h**) Cr 2p XPS of Cr-CoF-LDH before and after OER. (**i**) Theoretical model of H_2_O adsorbed on Co site in Cr-CoF-LDH and its adsorption energy. (**a**–**f**) Reproduced with permission [42]. Copyright 2020, Royal Society of Chemistry. (**g**–**i**) Reproduced with permission [43]. Copyright 2020, Elsevier.

The valence state of a single metal atom is also a regulator to tune the electrocatalytic performance of LDH materials. Wu’s group prepared the W doped Ni_3_Fe-LDH as the OER catalyst with high current densities of 100 and 1000 mA cm^−2^ at the low overpotentials of 247 and 320 mV, respectively [42]. In this material system, the W dopant with +6 valence state resulted in the negative shift of 1.1 eV for Ni and positive shift of 1.3 eV for Fe in W-Ni_3_Fe-LDH compared to pure Ni_3_Fe-LDH (Figure 3a–c). Such a shift indicated Ni being of a low valence state and Fe being of a high valence state in W-Ni_3_Fe-LDH, which were favorable for high OER activity with high current density (Figure 3d). In detail, the OER activity depends on the content of W dopant as presenting the first increase of OER activity and then a decrease (Figure 3e). The excessive content of W would lead to the increased charge-transfer resistance, which was the main reason for the decreased OER activity of W-Ni_3_Fe-LDH with a high amount of W dopant. In the long-time test, W-Ni_3_Fe-LDH exhibited superior stability in the continuous 50 h test (Figure 3f). Such a dopant regulating engineering strategy to enhance the OER activity is also demonstrated by the Cr dopant in CoFe-LDH [43]. As is well known, electrochemical activation is a general process before obtaining the electrocatalytic test. After the OER test, the Cr and Co species in Cr-CoFe-LDH material exhibited a higher valence state compared to pure CoFe-LDH (Figure 3g,h), which indicated that the formed Co and Cr species with high valence states are the highly active catalytic sites for OER. DFT calculation exhibited that the Co site in Cr-CoFe-LDH material possessed a strong ability of H_2_O dissociation with a short chemical bond between H and OH in H_2_O compared to other adsorption configurations over Cr and Fe sites (Figure 3i), thus greatly enhancing the reaction of water splitting into OH* that was favorable for O_2_ generation. Recently, kundu’s group reported that Ce as the dopant incorporated into NiCo-LDH to realize the goal of greatly enhancing OER activity [44]. The Ce dopant generated a higher ratio of Ni^3+^ species to Ni^2+^ in Ce-NiCo-LDH compared to pure NiCo-LDH, indicating that more Ni^3+^ sites existed in NiCo-LDH, thus increasing the number of highly active catalytic sites of Ni^3+^ as the OER catalytic center. Nowadays, more and more non-precious metal doped LDH have been developed as superior electrocatalysts in water splitting, such as W-Ni(OH)_2_/NiOOH [45], Mo-NiCo-LDH [46], Fe-NiCo-LDH [47], and Ta-FeNi-LDH [48]. Due to the plentiful non-precious metals and its amount of regulation engineering on electrocatalytic performance, a non-precious metal doping strategy provides a vast opportunity to develop excellent electrocatalysts in water splitting.

### 2.3. Non-Metal Doping

Apart from the metal doping enhancing the electrocatalytic performance of LDH, non-metal atom dopant also can form highly active catalytic sites due to their different electronegativities compared to oxygen atoms [49,50], such as F, S, P, and B [51,52,53,54]. These non-metal atoms bonding with metal atoms induce the different electron states on metals compared to pure LDH and simultaneously generate some vacancy defects, which realize the tuned electron density of LDH aiming to optimize the adsorption energies of intermediates, thus exhibiting the enhanced electrocatalytic performance.

**Figure 4:**
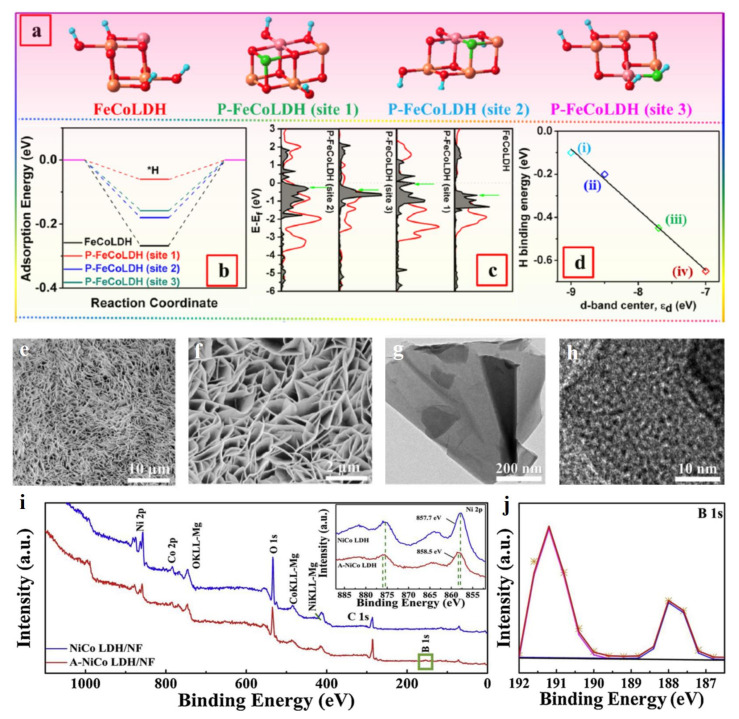
Theoretical studies and characterizations of non-metal atom doped LDH catalysts. (**a**) The theoretical structure models and (**b**) H* adsorption free energy of various sites over P-FeCoLDH. (**c**) Local density of states projected for the adsorbed H atom (H-DOS, dark shaded area). (**d**) The relationship of DFT-based hydrogen adsorption energies (ΔE) and metal *d*-band center positions on four different surficial sites, (**i**) P-FeCoLDH (site 1), (ii) P-FeCoLDH (site 3), (iii) P-FeCoLDH (site 2), and (iv) FeCoLDH. (**e**,**f**) SEM images of B-NiCo-LDH. (**g**,**h**) TEM images of B-NiCo-LDH. (**i**) Full XPS spectrum of B-NiCo-LDH. (**j**) B 1s spectrum of B-NiCo-LDH. (**a**–**d**) Reproduced with permission [51]. Copyright 2021, Elsevier. (**e**–**j**) Reproduced with permission [52]. Copyright 2020, Elsevier.

Tang et al. prepared P doped FeCo-LDH (P-FeCoLDH) supported on Cu foam using NaH_2_PO_4_ as a precursor in heat treatment at 350 °C [51]. P dopant could increase the electrochemical catalytic surface area (ECSA) and conductivity of FeCo-LDH, which significantly enhanced the number of catalytic sites and facilitated the electron transfer process during HER, respectively. Simultaneously, P-FeCoLDH possessed a lowest ΔG_H*_ (−0.27 eV) value, indicating its best H* adsorption state which facilitated H_2_ formation. The best H* adsorption state of P-FeCoLDH resulted from the tuned *d*-band center near to the anti-bonding orbital σ* of H* species. Because of a lower electronegativity for P compared to O, P-FeCoLDH exhibited a lower static contact angle in the water, which indicated that P atoms facilitated the adsorption of the H_2_O molecule, thus promoting the dissociation of water (Figure 4a-d). This phenomenon is also demonstrated by B doped NiCo-LDH (B-NiCo-LDH) to realize the high catalytic performance of HER with a low overpotential of 381 mV at a high current density of 1000 mA cm^−2^, which is better than that of the Pt/C catalyst [52]. B atom could be introduced into LDH lattice in the form of borate (BO_3_ species), which led to the generated amorphous NiCo-LDH. Such a newly formed phase would generate a large number of defects, such as *V*_O_, unsaturated atoms, more exposed active sites, and enhanced electrical conductivity (Figure 4e-j). Meanwhile, the existing BO_3_ species easily reacted with hydroxyl in alkaline electrolyte to form four-coordinated BO_4_. The formed BO_4_ groups with strong proton accepting ability promoted the dissociation of adsorbed H_2_O molecule through adsorbing H atoms and then transferred them onto the nearby metal atoms. H_2_ molecules could be released after two H atoms combining with each other over metal atoms.

**Figure 5:**
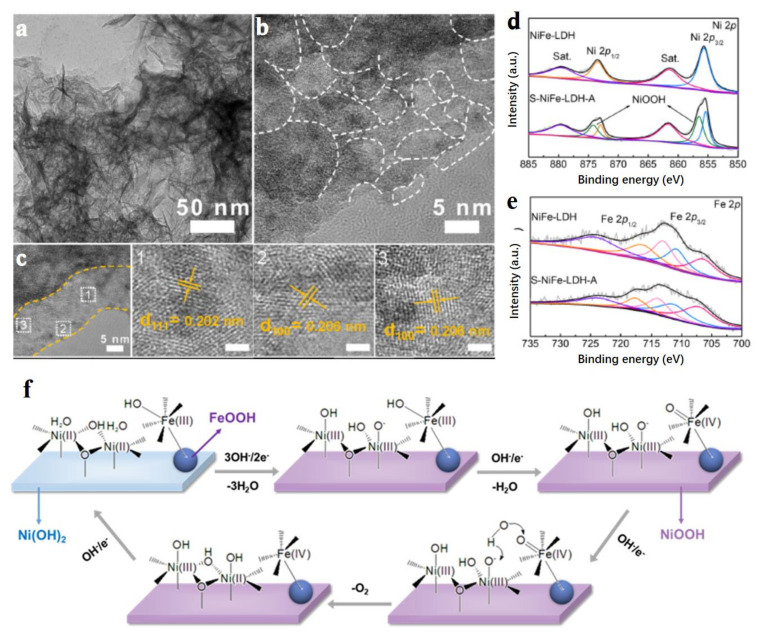
Characterization and OER mechanism of S-NiFe-LDH. (**a**) TEM image. (**b**,**c**) High resolution (HR) TEM image. (**d**) Ni 2p XPS spectra of S-NiFe-LDH and NiFe-LDH. (**e**) Fe 2p XPS spectra of S-NiFe-LDH and NiFe-LDH. (**f**) OER mechanism over Fe and Ni sites in S-NiFe-LDH. (**a**–**f**) Reproduced with permission [53]. Copyright 2021, Elsevier.

Non-metal atoms doping is also an efficient method to enhance the OER activity of LDH. Chai’s group prepared S doped NiFe-LDH (S-NiFe-LDH) on MoNi foam support by the electrochemical metallic deposition method in a thioacetamide (TAA) solution [53]. In this system, TAA provided the S dopant incorporated into NiFe-LDH during the deposition; meanwhile the released S^2−^ species reacted with Ni^2+^ and Fe^2+/3+^ to generate more exposed edges of LDH. In this preparation process, the S-NiFe-LDH featured with the properties of having a large active surface area, more exposed active sites, and long-term robustness (Figure 5a–c), jointly contributing to high the OER performance, including a low overpotential of 270 mV at 50 mA cm^−2^ and favorable durability. S atoms incorporated into NiFe-LDH facilitated the formation of high valence states of Ni and Fe (Figure 5d,e), which were highly active catalytic sites for OER with favorable process of O species transfer cycling. This corrosive condition for preparing LDH is also reflected in the F doped Co_3_Fe-LDH system [54]. Wang et al. synthesized F doped Co_3_Fe-LDH (F-Co_3_Fe-LDH) by the CHF_3_-plasma etching method. During the CHF_3_-plasma process, F atoms replacing the O atoms and the metal vacancies (*V*_M_) generated at the same time. Simultaneously, the generated O atoms would also result in a strong interaction between Co and Fe atoms with an octahedral coordination, thus forming highly active catalytic sites for OER with a low energy barrier. The F atom attracted more electrons from metal sites due to its high electronegativity being 4.0, which resulted in higher oxidation states of Co and Fe compared to pure Co_3_Fe-LDH, as demonstrated by XPS and XANES spectra. As is well known, the Co (Ni, Fe etc) metallic sites with high valence state possess the highly intrinsic OER activity [55,56]. Therefore, F-Co_3_Fe-LDH exhibited a low overpotential of 276 mV at 10 mA cm^−2^ due to the optimized free energy profile for OER from the F dopant and more exposed active sites. Nowadays, the doping with nonmetallic elements has been regarded as an efficient approach to improve the electrocatalytic performance of LDH by the utilization of their different electronegativities compared to O [57,58], which mainly generated *V*_O_, *V*_M_, unsaturated atoms, exposed more active sites, and enhanced electrical conductivity, as well as forming new high-catalytic sites with low theoretical energy barriers for HER and OER.

## 3. Heterojunction Strategy

It is an efficient approach to enhance the electrocatalytic performance of LDH by constructing the heterojunction of LDH with other compounds. In this kind of material system, new highly active catalytic sites can be formed in the interface between two compounds due to their strong chemical interaction, which generates new chemical bonds and tunes the electronic state of individual compounds. Generally, there exist two functions of heterojunction for realizing the high electrocatalytic performance of LDH-based catalysts. Firstly, the enhanced conductivity of LDH-based materials can be obtained through hybridizing with high conductive metal compounds, which efficiently improve the electron transfer rate between reactants and catalysts, thus greatly facilitating the reaction dynamics. Secondly, new catalytic sites are formed between the two compounds, which can optimize the free energy profiles of reactions, thus lowering the overpotentials. In this section, we will summarize the recent progress in the heterojunction strategy for constructing LDH-based catalysts hybridized by other compounds, including metals, metal compounds, carbon, and organic materials, which also involves their preparation methods, characterizations, electrocatalytic performances, and theoretical studies.

### 3.1. Metals

**Figure 6:**
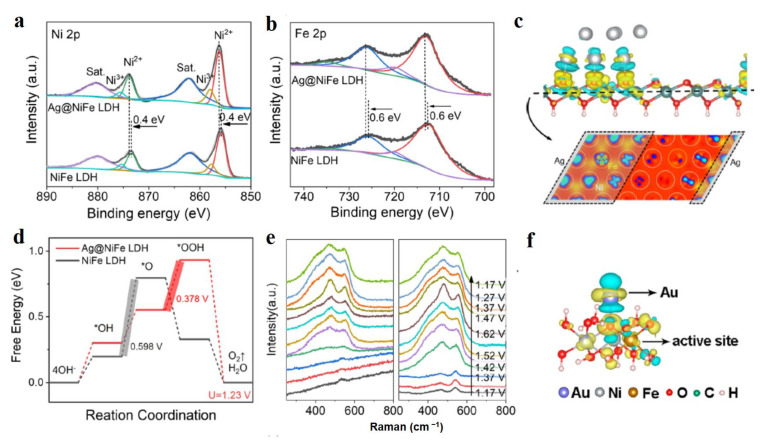
Characterizations and theoretical studies of heterojunction for LDH materials. (**a**,**b**) Ni 2p (**a**) and Fe 2p (**b**) XPS spectra for Ag@NiFe-LDH. (**c**) Side view (up) and 2D view (bottom) of differential charge density of Ag@NiFe-LDH. (**d**) OER energy profiles of NiFe-LDH and Ag@NiFe-LDH. (**e**) *In suit* Raman spectra of NiFe-LDH and Ag@NiFe-LDH. (**f**) Differential charge densities of NiFe-LDH with Au atom when one O atom is adsorbed on the Fe site. Iso-surface value is 0.004 eÅ^−3^. Yellow and blue contours represent electron accumulation and depletion, respectively. (**a**–**e**) Reproduced with permission [59]. Copyright 2022, Elsevier. (**f**) Reproduced with permission [63]. Copyright 2018, American Chemical Society.

Metals and their alloys possess excellent conductivity, which not only greatly improves the electron transfer during reactions over LDH-based materials, but also forms the new highly catalytic centers with low overpotentials. Nowadays, the metals and their alloys hybridized with LDH are mainly precious metals, such as Ag, Pt, Au, and PtNi alloy [59,60,61,62,63,64,65]. Of course, some non-precious metals also can form a heterojunction with LDH, for example, CuNi alloy [66]. Song et al. skillfully combined the negatively charged Ag nanoparticles with positively charged different kinds of hydroxides (NiFe-LDH, CoFe-LDH, Co(OH)_2_, Ni(OH)_2_) through the electrostatic adsorption principle to construct advanced OER electrocatalysts [59]. In these Ag nanoparticles modified LDH materials, Ag-NiFe-LDH exhibited the best OER activity with an overpotential of 246 mV at 10 mA cm^−2^, as well as a long-time stability in the water splitting cell at 500 mA cm^−2^ in a continuous 110 h test. Ag nanoparticles resulted in the increase of the valence state for Ni and Fe in Ag-NiFe-LDH (Figure 6a,b), indicating the strong chemical interaction between Ag nanoparticles and LDH, and demonstrated as well by the differential charge densities over Ni, Fe, and Ag elements (Figure 6c). Ni species with high valence states of +3 are widely regarded as highly active catalytic sites for OER with low theoretical overpotentials of OER energy profile (Figure 6d), which is the main result of the high OER performance of Ag-NiFe-LDH. In suit Raman spectra exhibited that the new peak at 549 cm^−1^ assigned to Ni^3+^-O species appeared at a low overpotential of 1.42 V while the stretching vibration of Ni^2+^-OH (452 cm^−1^) and Ni^2+^-O (531 cm^−1^) diminished over Ag-NiFe-LDH (Figure 6e), indicating that Ag nanoparticles facilitated the Ni^2+^/Ni^3+^ oxidation with a lower applied potential compared to NiFe-LDH, thus leading to high OER activity. Meanwhile, Ag-NiFe-LDH material possessed a 0.378 eV energy barrier in the limited step of O* to OOH* during OER, much lower than that of 0.598 eV from OH* to O* over NiFe-LDH, which indicated that Ag hybridized NiFe-LDH successfully formed the highly-catalytic sites for OER. The newly formed catalytic site was also demonstrated by the appearance of electron states of Ni and Fe near the Fermi level. Metal nanoparticles generating the high valence state of atoms on LDH are also well demonstrated by the Pt clusters on NiFe-LDH [60]. Generally, the newly formed high species of atom between LDH materials and metals usually possess the optimized energy profiles of electrocatalytic reactions [61]. Sun et al. used a layered triboelectric nanogenerator (TENG) as a power source to generate a pulsed direct current with high voltage to electrodeposit Pt nanoclusters of 2 nm onto NiFe-LDH nanosheets (Pt-NiFe-LDH) [62]. The as-synthesized Pt-NiFe-LDH electrocatalyst exhibited superior electrocatalytic activities to NiFe-LDH loaded with Pt nanoparticles prepared by the traditional NaBH_4_ reduction method. The current density of 50 mA cm^−2^ could be reached at the ultra-low overpotential of 86 mV for HER. By controlling the working frequency and deposition time of layered TENG, the size of Pt nanoclusters was easily tuned. The high electrocatalytic performance of Pt-NiFe-LDH was attributed to the synergistic effect between Pt nanoclusters and NiFe-LDH which greatly promoted the fracture of HO-H and optimized the adsorption energies of intermediates. This unique strategy of preparing heterojunction electrocatalyst easily tuned the valence state of metal atoms in LDH making partial Fe^3+^ into Fe^2+^, and thus realized the electronic regulation. Considering the high cost of noble metal electrocatalysts, Zhang’s group prepared nano-sized Au atom modified NiFe-LDH (Au-NiFe-LDH) to reduce the cost of electrocatalysts and improve the utilization efficiency of metal atoms [63]. The prepared catalyst delivered a six times higher current density at an overpotential of 280 mV than that of NiFe-LDH, which resulted from the new formed Fe sites constructed by single Au atoms (Figure 6f). Because of this point, the reaction step of O* to OOH* could be optimized, thus exhibiting superior OER activity over Au-NiFe-LDH.

**Figure 7:**
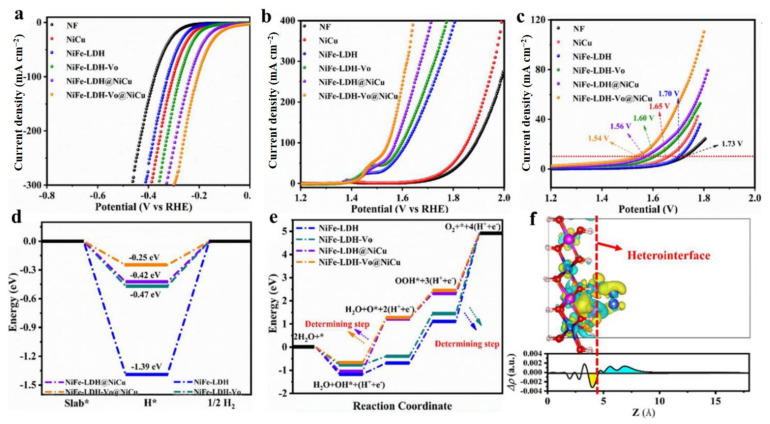
Electrocatalytic performance and theoretical studies of NiFe-LDH-Vo-NiCu. (**a**) LSV curves of HER. (**b**) LSV curves of OER. (**c**) LSV curves in water splitting cell. (**d**) Calculated free energy diagram of HER. (**e**) Calculated free energy diagram of OER intermediates at zero potential (U = 0). (**f**) The differential charge density diagram of NiFe-LDH-Vo-NiCu diagram. (**a**–**f**) Reproduced with permission [66]. Copyright 2022, Elsevier.

Apart from the above analyzed precious metal nanoparticles/clusters that can form the heterojunction to enhance the electrocatalytic performance of LDH, transition metals (Ni, Cu and their alloys) with low cost have been reported as substitutes for precious metals to hybridize with LDH to form the heterojunction. Lei et al. reported that NiFe-LDH modified by NiCu alloy exhibited an excellent electrocatalytic performance in the water splitting cell [66]. Firstly, NiFe-LDH was treated with H_2_O_2_ at 120 °C for 6 h to generate oxygen vacancies (*V*_O_). Secondly, the electrodeposition method was performed at a negative potential to construct NiFe-LDH-NiCu heterojunction. The *V*_O_ generated by H_2_O_2_ etching effectively tuned the electronic structure of the composites and exposed more active sites. NiFe-LDH-*V*o-NiCu delivered the current density of 50 mA cm^−2^ with only 166 mV overpotential for HER and a current density of 50 mA cm^−2^ with only 244 mV overpotential for OER (Figure 7a,b). In a water splitting cell under alkaline conditions, NiFe-LDH-*V*o-NiCu acted as both cathode and anode catalysts, exhibiting the current density of 10 mA cm^−2^ at 1.54V (Figure 7c). Due to the generation of oxygen vacancies and the deposition of NiCu, the electronic structure of NiFe-LDH was well tuned. DFT studied showed that NiFe-LDH-*V*o-NiCu had a value of |∆G_H*_| near zero, indicating that the adsorption energy of H* for NiFe-LDH was successfully optimized by the introduction of *V*_O_ and NiCu alloy (Figure 7d). For OER, the existence of *V*_O_ and NiCu optimized the energy profile of OER during the reaction as presenting the lowest energy barriers (Figure 7e). According to Bader’s charge density, the most intense electron movement occurred at the coupling interface between NiFe-LDH and NiCu, and the electrons of NiFe-LDH were captured by NiFe (Figure 7f). Nowadays, metals and their alloys hybridized with LDH have been widely applied in constructing advanced electrocatalysts due to their convenient methods.

### 3.2. Metal Compounds

**Figure 8:**
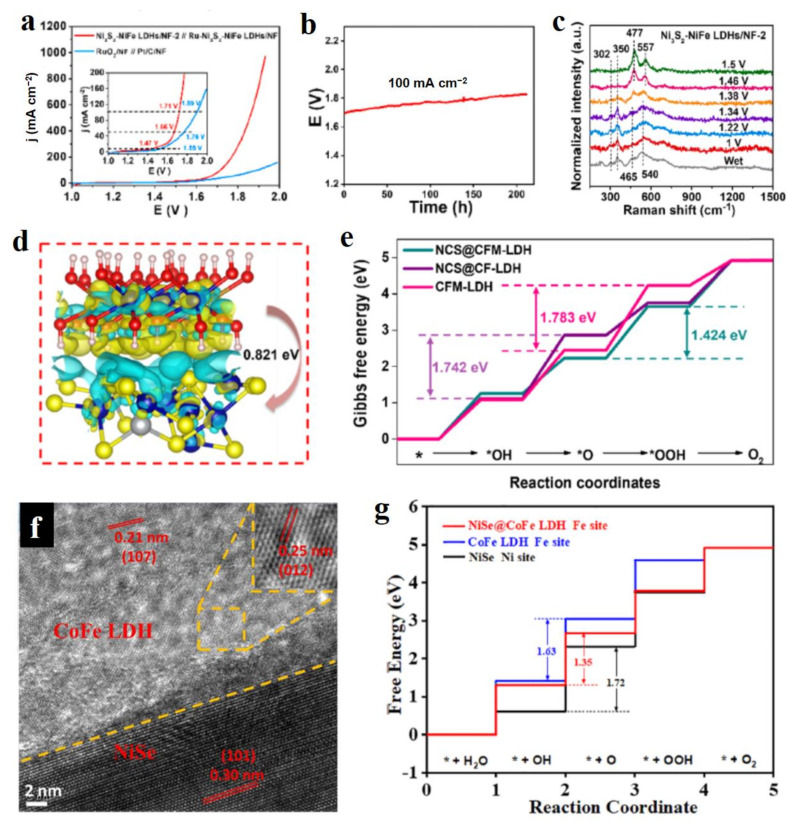
Electrocatalytic performance and theoretical studies of LDH hybridized with metal sulfides and selenides. (**a**) LSV curves of water splitting cell over Ni_3_S_2_-NiFe-LDHs. (**b**) Stability of Ni_3_S_2_-NiFe-LDHs in water splitting cell. (**c**) Raman spectra in water splitting cell using Ni_3_S_2_-NiFe-LDHs as catalyst. (**d**) Charge density difference at the NiCo_2_S_4_-CoFe-LDH interface, and the charge depletion and accumulation are in yellow and blue, respectively. (**e**) Calculated free energy diagram of OER. (**f**) TEM images of NiSe-CoFe-LDH. (**g**) Calculated free energy diagram of OER. (**a**–**c**) Reproduced with permission [67]. Copyright 2022, Elsevier. (**d**,**e**) Reproduced with permission [70]. Copyright 2022, Elsevier. (**f**,**g**) Reproduced with permission [73]. Copyright 2022, Elsevier.

Due to the plentiful combination modes of metal and non-metal atoms, a large number of heterojunctions constructed by LDH and metal compounds have been developed as water splitting electrocatalysts, including metal oxides, chalcogenides, phosphides, and carbides. These metal compounds featured with their unique electronic structures have been widely utilized as efficient regulators to enhance the electrocatalytic performance of LDH materials. Li et al. reported a novel Ni_3_S_2_-embedded NiFe-LDH heterogeneous structured porous nanosheet on Ni foam (Ni_3_S_2_-NiFe-LDHs/NF), which was synthesized via a simple one-pot solution method at room temperature for only 15 min [67]. In this preparation process, NaHS played a vital role in synthetizing this material. It not only reacted with metal cations to produce sulfides, but also hydrolyzed to produce OH^−^, which provided the condition for the growth of LDH. The as-prepared catalyst possessed rich coupling interfaces and an enhanced charge transfer capability, as well as the porous nanosheet structure endowed with its more exposed active sites, which jointly contributed to excellent OER activity. In detail, the current densities of 50, 500, and 1000 mA cm^−2^ could be realized at the low overpotentials of 230, 285, and 303 mV, respectively. Furthermore, the water splitting cell constructed by Ni_3_S_2_-NiFe-LDHs/NF exhibited the high current densities of 100 and 500 mA cm^−2^ at low voltages of 1.71 and 1.85 V, respectively, which is much better than that of the cell formed by Pt/C and RuO_2_ (Figure 8a), and also possessed excellent stability in more than 200 h of continuous testing (Figure 8b). In this work, the authors used in situ Raman spectra to investigate the dynamic surface chemistry of Ni_3_S_2_-NiFe-LDHs. With the increase of applied potential during OER, the characteristic Raman peaks of Ni_3_S_2_ at 302 and 350 cm^−1^ were gradually decreased and then disappeared at 1.46 V vs. RHE, while the Raman peaks of 477 and 557 cm^−1^ assigned to Ni^3+^-O vibrations emerged and were transferred from 465 and 540 cm^−1^ assigned to Ni^2+^-O (Figure 8c). This result demonstrated well that the Ni^3+^ species was the highly active catalytic site for OER, and the Ni_3_S_2_ was not stable and easily transformed into metal hydroxides. Feng et al. reported a unique cactus-like morphology of the NiCo_2_S_4_-NiFe LDH heterostructure, which performed long-term stability and better OER activity than commercial IrO_2_ [68]. The enhanced OER activity was attributed to the strong interaction between NiCo_2_S_4_ and NiFe-LDH. In addition, NiFe-LDH with a nano-sheet and nano-wire structure inhibited the collapse of NiCo_2_S_4_ hollow nanospheres well, and thus protected NiCo_2_S_4_ from chemical corrosion to ensure its super stability. Nowadays, many other metal sulfides have been reported as components to hybridize with LDH for developing excellent electrocatalysts, such as FeNi_2_S_4_ [69], NiCo_2_S_4_ [70], and NiS_x_ [71]. Generally, this kind of heterojunction exists as the clear electron transfer process between the individual components, which greatly optimizes the energy profiles of reactions, thus leading to low overpotentials. For instance, electrons accumulated at the interface between CoFe-LDH and NiCo_2_S_4_ (Figure 8d), thus forming the highly active catalytic sites for OER with an optimized energy profile (Figure 8e) [70]. For OER, more electron states accumulated over the Fermi level and facilitate the reaction with reactants due to the fast electron transfer process, which is the guidance for the design of OER materials towards high electrocatalytic activity.

Similar to the case of metal sulfides, metal selenides also hybridize with LDH to form high performance heterojunction catalysts. Peng et al. prepared FeNi_2_Se_4_-FeNi-LDH heterostructure by the in-situ growth of FeNi-LDH nanosheet array on a nickel foam conductive substrate and a subsequent partial selenization treatment, which performed the low overpotentials of 205 mV for OER and 106 mV for HER at a current density of 10 mA cm^−2^ [72]. In the water splitting cell, FeNi_2_Se_4_-FeNi-LDH acted as a bifunctional catalyst to drive the cell in a voltage of 1.56 V at 10 mA cm^−2^, which is lower than that of the cell constructed by Pt/C and RuO_2_ (1.58 V), indicating its great potential in substituting for precious metal catalysts. NiSe-CoFe-LDH nanoarrays were prepared by the first selenization method and then an electrodeposition path [73]. Due to the intimate contact of NiSe and CoFe-LDH (Figure 8f), the electronic interaction between NiSe and CoFe-LDH was easily observed in XPS spectra. Such interaction resulted in the formed highly active catalytic sites for OER with low theoretical overpotentials (Figure 8g). Metal selenides hybridized with LDH have increasingly demonstrated that LDH can strongly interact with metal selenides to form highly active catalytic sites, which are also an efficient candidate for improving the electrocatalytic performance of LDH, e.g., Cu_2_Se-NiFe-LDH nanosheets [74], NiSe-FeNi-LDH [75].

**Figure 9:**
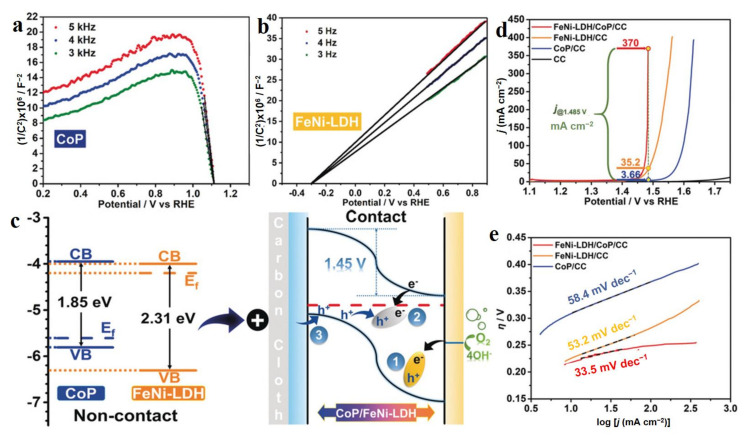
Characterizations and electrocatalytic performance of CoP-FeNi-LDH junction. (**a**) Mott–Schottky plots of CoP. (**b**) Mott-Schottky plots of FeNi-LDH. (**c**) The energy diagrams of CoP and FeNi-LDH and the proposed mechanism for electrocatalyzing OER in the FeNi-LDH-CoP p-n junction under the condition of applied potential. (**d**) LSV curves of OER over serial catalysts. (**e**) Tafel slopes of catalysts. (**a**–**e**) Reproduced with permission [77]. Copyright 2019, Wiley-VCH.

Metal phosphides (MP_x_) have been widely applied in electrocatalysis due to their multifunctional active sites, tunable structures, and compositions, as well as plentiful electronic properties [76]. Liu et al. reported a FeNi-LDH-CoP n-p junction structure composed of amorphous n-type FeNi-LDH and p-type CoP nanoarrays, which exhibited excellent OER performance with 231 mV overpotential at 20 mA cm^−2^ [77]. From Mott–Schottky (M-S) plots of FeNi-LDH and CoP, it was easily observed that FeNi-LDH was a n-type semiconductor with a positive slope of Tauc plot and CoP was a p-type semiconductor with a negative slope of Tauc plot (Figure 9a,b) The flat band (FB) potentials for FeNi-LDH and CoP were −0.3 and 1.11 V vs. RHE, respectively. Therefore, the band structures of FeNi-LDH and CoP could be obtained, as shown in Figure 9c (left), when they were not contact. Due to the different Fermi levels of two semiconductors, electrons over CoP transferred to FeNi-LDH when they were in contact (Figure 9c (right)). The electron transfer process could be easily observed in XPS data of serial catalysts, which led to enhanced OER activity of CoP-FeNi-LDH with a low Tafel slope of 33.5 mV dec^−1^ (Figure 9d,e). The excellent OER performance of the CoP-FeNi-LDH junction was attributed to the positively charged FeNi-LDH site possessing a strong ability for adsorbing OH^−^, thus greatly facilitating the OER dynamics with a low energy barrier. This work provides an efficient approach to develop advanced electrocatalysts through constructing an n-p and p-p junction with a strong ability for OH^−^ adsorption [78,79]. In short, metal phosphides hybridized with LDH usually possess excellent electrocatalytic performance in water splitting with several hundred current densities up to 300 mA cm^−2^, especially for their superior OER activities [78,80,81].

**Figure 10:**
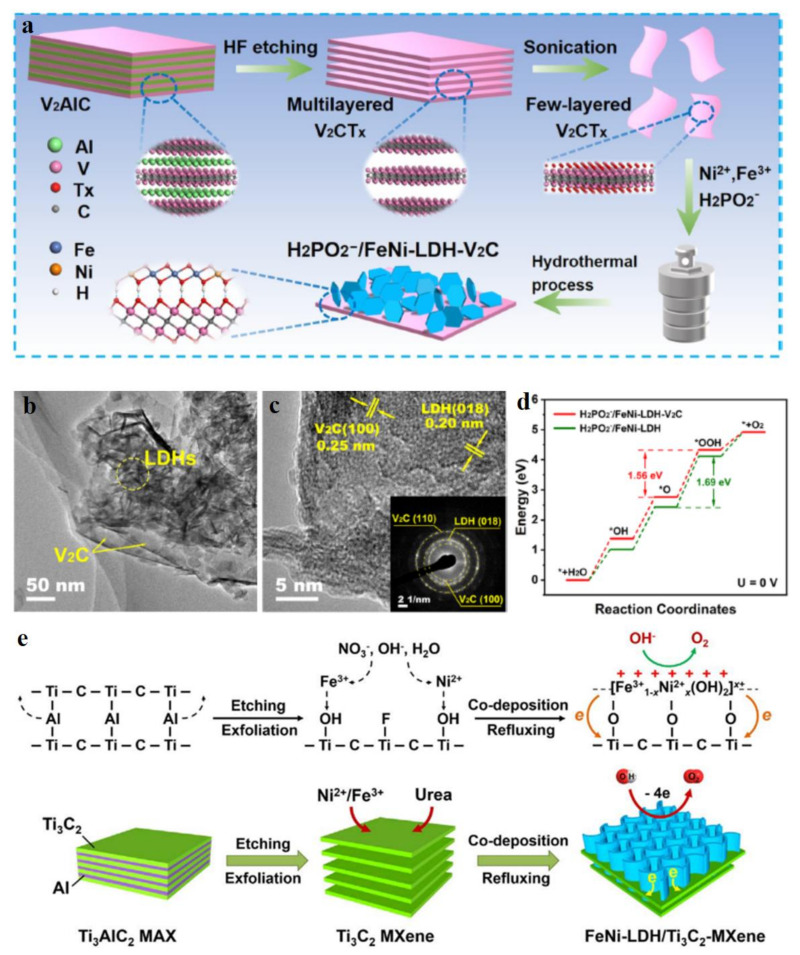
Schematic illustration, characterizations, and electrocatalytic performance of MXene hybridized with LDH. (**a**) Preparation process of H_2_PO_2_^−^/NiFe-LDH-V_2_C. (**b**,**c**) TEM images of H_2_PO_2_^−^/NiFe-LDH-V_2_C. (**d**) Calculated energy profile of OER over H_2_PO_2_^−^/NiFe-LDH-V_2_C and H_2_PO_2_^−^/NiFe-LDH at U = 0 V. (**e**) Preparation process of NiFe-LDH-Ti_3_C_2_. (**a**–**d**) Reproduced with permission [84]. Copyright 2021, Elsevier. (**e**) Reproduced with permission [85]. Copyright 2018, Elsevier.

In recent years, two-dimensional (2D) metal carbides and nitrides (MXenes) as promising electrocatalysts in the energy conversion field, have achieved more and more attention from researches due to their high specific surface area, good electrical conductivity, superior mechanical strength, and tunable surface chemistry [82]. Metal carbides and nitrides have also been well demonstrated as efficient candidates to hybridize with LDH aiming to obtain high performance electrocatalysts [82,83]. Hou et al. coupled a few-layer V_2_C nanosheets and FeNi LDH nanosheets with H_2_PO_2_^−^ intercalation to form H_2_PO_2_^−^/NiFe-LDH-V_2_C hybrid material for enhancing OER with 250 mV overpotential at 10 mA cm^−2^ and a small Tafel slope of 46.5 mV dec^−1^ [84]. This heterojunction was prepared by combining the chemical etching and hydrothermal method (Figure 10a). Due to the 2D morphology structures of V_2_C and NiFe-LDH materials, the H_2_PO_2_^−^/NiFe-LDH-V_2_C possessed intimate contact between the individual components (Figure 10b,c), which led to electron transfer between them, as demonstrated by the shifts of their elements’ XPS and XAS spectra. Such electron transfers generated the new highly catalytic OER sites with optimized energy profiles with a theoretical overpotential of 1.56 eV, much lower than that of H_2_PO_2_^−^/NiFe-LDH, being 1.69 eV (Figure 10d). In this material system, the H_2_PO_2_^−^ acted as an anion intercalation to adjust the intrinsic electronic structure of pristine NiFe-LDH, which also played a synergistic role in enhancing OER activity, much better than that of CO_3_^2−^ reference. DFT results showed that the H_2_PO_2_^−^/NiFe-LDH-V_2_C heterojunction owned a higher electron state than that of H_2_PO_2_^−^/NiFe-LDH, which facilitated the electron transfer process during OER, thus leading to a low energy barrier. Qiu et al. developed a hierarchical NiFe LDH/Ti_3_C_2_-MXene nanohybrid as an efficient OER catalyst [85]. Ti_3_C_2_ was synthesized by etching Ti_3_AlC_2_ with LiF and HCl to form Ti_3_C_2_ with 2D morphology structure and plentiful superficial OH^−^ chemical groups. For NiFe-LDH growth, Ni^2+^/Fe^3+^ tended to combine with OH- groups on Ti_2_C_3_ due to the electrostatic adsorption, and then the subsequent hydrolysis and oxidation of MO_6_ groups provided support for the growth of LDH on Ti_2_C_3_ (Figure 10e). In this process, LDH could be firmly anchored on the surface of Ti_2_C_3_, efficiently preventing its agglomeration, thus endowing its excellent stability during OER. Meanwhile, the strong interaction between them promoted the oxidation-reduction process of LDH for OER and effectively electron transferred between the NiFe-LDH and Ti_2_C_3_ due to the high conductivity of Ti_2_C_3_. Generally, two-dimensional metal carbides and nitrides are the big family materials to hybridized with LDH materials due to their tunable and plentiful surficial chemical groups which provide the ideal sites for LDH growth with uniform dispersion. Simultaneously, the high conductive properties of metal carbides and nitrides endows the fast electron transfer between the reactants and catalytic sites, thus facilitating the dynamics of reactions during electrocatalysis. Therefore, increasing MXenes-LDH heterojunction catalysts have been reported as excellent electrocatalysts in water splitting [82,83,86,87,88], especially for their superior OER performance.

**Figure 11:**
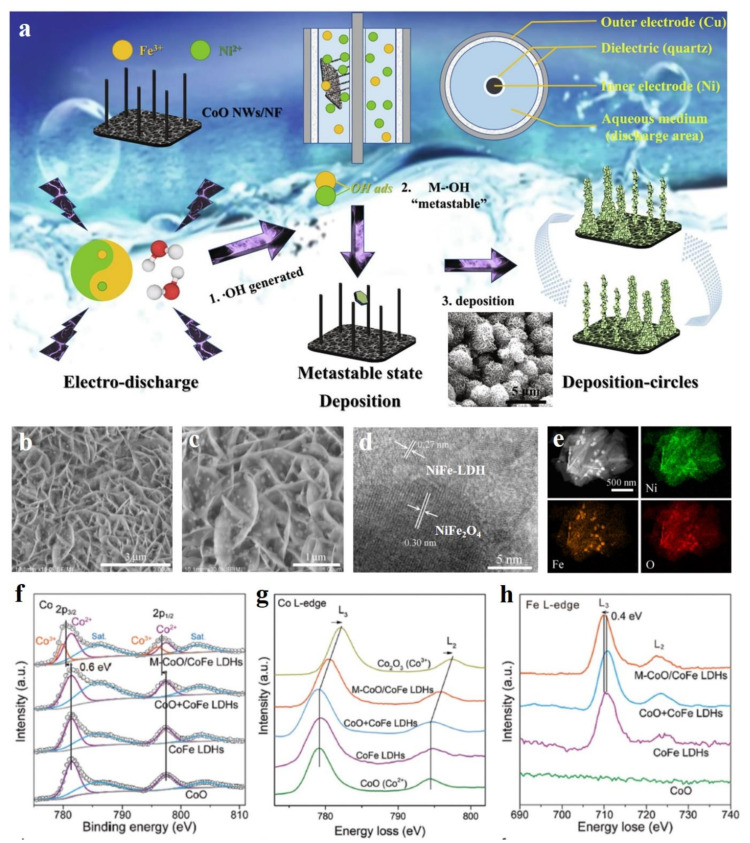
Schematic illustration and characterizations of metal oxides hybridized with LDH. (**a**) Schematic illustration of preparing CoO-NiFe-LDH. (**b**,**c**) SEM images of NiFe_2_O_4_/NiFe-LDH. (**d**) High resolution TEM image of NiFe_2_O_4_/NiFe-LDH. (**e**) Dark field TEM and elemental mapping images of NiFe_2_O_4_/NiFe-LDH. (**f**) Co 2p XPS spectra of serial Co-based materials. (**g**) Co EELS spectra of serial Co-based materials. (**h**) Fe EELS spectra of serial Co-based materials. (**a**) Reproduced with permission [90]. Copyright 2021, Elsevier. (**b**–**e**) Reproduced with permission [91]. Copyright 2018, American Chemical Society. (**f**–**h**) Reproduced with permission [92]. Copyright 2018, Wiley-VCH.

Transition metal oxides, featured by their low cost, high stability, and excellent electrocatalytic activity, also have been candidates for hybridizing with LDH to construct excellent catalysts [89]. Huang et al. prepared CoO-NiFe-LDH/NF electrocatalysts with a 3D open structure via a novel dielectric barrier discharge (DBD) microplasma [90]. This unique preparation method possesses the three advantages in guiding material formation (Figure 11a): (1) due to the higher plasma chemical reactivity, BDB microplasma had a higher density of free radicals, which was favorable for the formation of •OH in the aqueous solution of the BDB reactor; (2) the integration of electric work endowed the metal ions and OH in the aqueous solution with enough energy to keep them in a “metastable state” to ensure the nucleation and growth of NiFe-LDH; (3) and cyclic deposition can ensure that NiFe LDH was uniformly distributed on CoO/NF. The unique 3D open structure of CoO-NiFe-LDH/NF was favorable for reactant diffusion and product release, thus exhibiting an enhanced OER activity. Gao et al. used one-step solvothermal method to prepare the NiFe_2_O_4_ nanoparticles loaded on NiFe-LDH nanosheets. NiFe_2_O_4_ nanoparticles were uniformly dispersed on the NiFe-LDH nanosheets (Figure 11b–e), which provided more exposed actives for OER [91]. In details, the large current densities of 500 and 1000 mA cm^−2^ for OER could be obtained under the small overpotentials of 242 and 265 mV, respectively, as well as long time stability in a continuous 20 h test under the current density of 500 mA cm^−2^. The superior electrocatalytic performance of NiFe_2_O_4_-NiFe-LDH was attributed to the strong interface coupling between NiFe_2_O_4_ and NiFe-LDH, which endowed the composite with better electron mobility and more catalytic active sites to participate in reactions. Strong chemical interactions between metal oxides and LDH have also been well supported on the CoO nanoclusters on CoFe-LDH support [92]. XPS and XAS spectra showed that the Co-O-Fe bond formed at the strong coupling interface of heterostructure contributed to the electron transfer from CoO to CoFe-LDH and the formation of a large amount of Co^3+^ (Figure 11f-h), thus leading to enhanced OER activity. Similarly, the NiO nanoparticles supported on NiFe-LDH nanosheets with intimate contact were also successfully prepared by the hydrothermal and post calcination method as efficient OER catalysts [93]. Zhao et al. used the -NH_2_ group to modify the surface of NiFe-LDH, in which the -NH_2_ group provided the sites for Ir^4+^ adsorption and IrO_2_ growth [94]. Due to this point, IrO_2_ nanoparticles with a uniform size of 5 nm were loaded on NiFe-LDH, thus leading to a low overpotential of 274 mV at a current density of 10 mA cm^−2^, a low Tafel slope of 59 mV dec^−1^, and superior stability in the continuous 35 h test. Nowadays, metal oxides have been developed as the big family candidates for constructing heterojunctions with LDH, e.g., CuO-CoOOH [95], CuO-FeCoNi-LDH [96], and Co_3_O_4_-FeOOH [97]. In these heterojunctions, the electron transfer between the individual components is the main reason for their high electrocatalytic performance, and the heterojunctions are usually the p-n type [95,96]. Recently, carbonate hydroxides have been reported as another component to hybridize with LDH materials to form high-performance OER and HER materials [98], which also opens other methods for modifying LDH materials by using metal carbonate hydroxides.

### 3.3. Carbon Materials

**Figure 12:**
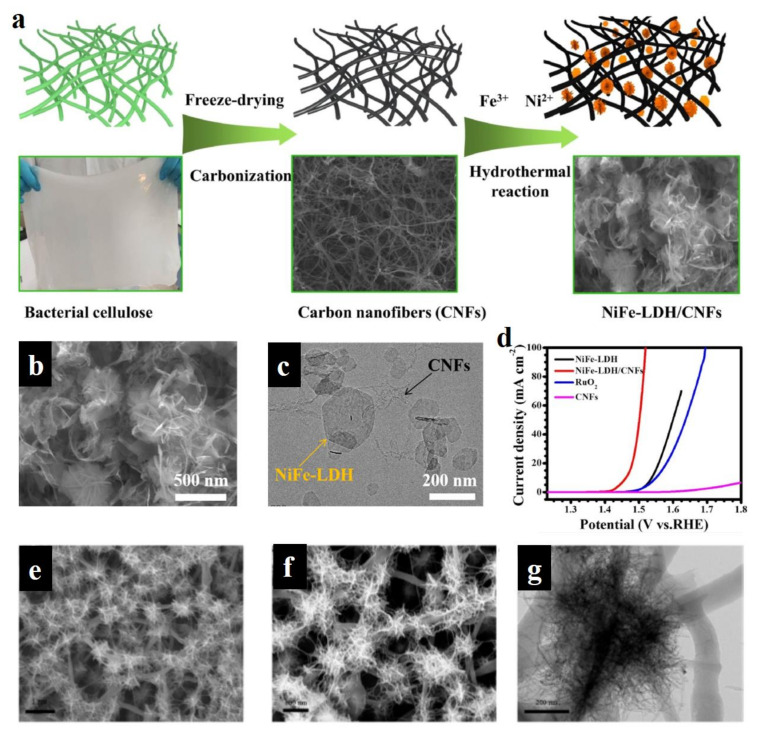
Schematic illustration, characterizations, and electrocatalytic performance of carbon materials hybridized with LDH. (**a**) Schematic illustration of preparing FeNi-LDH/carbon nanofibers. (**b**,**c**) SEM (**b**) and TEM (**c**) images of FeNi-LDH/carbon nanofibers. (**d**) LSV curves of serial catalysts. (**e**–**g**) SEM (**e**,**f**) and TEM (**g**) images of NiFe-LDH/carbon fibers. (**a**–**d**) Reproduced with permission [104]. Copyright 2022, Elsevier. (**e**–**g**) Reproduced with permission [106]. Copyright 2022, American Chemical Society.

As one of the huge reserve resources, carbon-based materials have been widely applied in the field of energy conversion and storage, due to their high specific surface area, low cost, high conductivity, tunable chemical modification (e.g., heteroatom doping), and variable morphological features (e.g., 1D, 2D, and 3D morphologies) [99,100,101,102]. As is well known, the poor conductivity of LDH greatly restricts the electron transfer between the bulk catalysts and reactant species, thus leading to the high overpotential of reactions [103]. Carbon materials featured by high conductivity are widely regarded as efficient candidates for hybridizing LDH to construct excellent electrocatalysts. Simultaneously, their porous structure endows LDH with intimate contact with carbon materials, and also provides more active sites exposed in the electrolyte, and also facilitates reactant and product diffusion. Liu et al. used bacterial cellulose as carbon source to obtain carbon nanofibers by the carbonization method, and then FeNi-LDH were loaded on carbon nanofibers using the hydrothermal method (Figure 12a) [104]. From SEM and TEM images, as shown in Figure 12b,c, carbon nanofibers as the binder efficiently linked the FeNi-LDH, thus establishing the 3D morphological feature. These FeNi-LDH/carbon nanofibers exhibited superior OER activity (230 mV overpotential at 10 mA cm^−2^) to commercial RuO_2_ and pure FeNi-LDH materials (Figure 12d). This work demonstrates well that the conductivity of catalysts is important for enhancing their electrocatalytic performance. Xu et al. successfully assembled the Ni-based LDHs/graphene quantum dots (GQDs) heterojunction catalyst on the foam nickel conductive skeleton by employing the electrostatic adsorption principle [105]. Combined with the DFT theoretical calculation, XPS analysis, and OER test results, it could be revealed that the pristine electronic structure of the prepared heterojunction catalyst was changed due to the strong interaction of the heterojunction interface, and thus the adsorption energy of the reaction intermediates was optimized. The electrons over LDH transferred to GQDs, which increased the valence state of metal cations in LDH and improved the adsorption capacity of OH^−^ in order to ensure the charge balance in LDH. This work suggests that the valence state of LDH can be tuned by carbon materials, thus optimizing the adsorption energies of intermediates towards lowering reaction energy barriers.

The 3D morphology of NiFe-LDH/carbon fiber heterojunction was easily prepared by the first carbonization of polyacrylonitrile (PAN) and iron phthalocyanine (FePc) and second hydrothermal method [106]. NiFe-LDH was uniformly dispersed on carbon fibers with nanosheet morphology (Figure 12e–g). This morphological feature facilitated reactant diffusion, thus improving the OER dynamics. Now, heterojunction composed of carbon materials and LDH have been more and more developed in recent years, such as NiCo-LDH/GO-CNTs [107], NiCo-LDH/fullerene dots [108], FeNiOOH/carbon dots [109], and NiCo-LDH/graphite felt [110]. As one of the traditional carbon dots (CDs), fullerene quantum dot (FQD) has attracted attention from researchers. Wang et al. designed a three-dimensional layered structure on foamed nickel by a simple self-assembly strategy, which consisted of CoNi-LDH modified by FQDs. FQDs/CoNi-LDH/NF exhibiting excellent HER and OER properties was attributed to FQDs being anchored in the interlayer and surface of NiCo-LDH to accelerate electron and mass transport [108]. In water splitting cell, the prepared FQD/CoNi-LDH/NF catalyst could be used as a cathode and anode to drive the overall water-splitting reaction, and the cell voltage of 1.59 V could reach 10 mA cm^−2^.

### 3.4. Organic Materials

**Figure 13:**
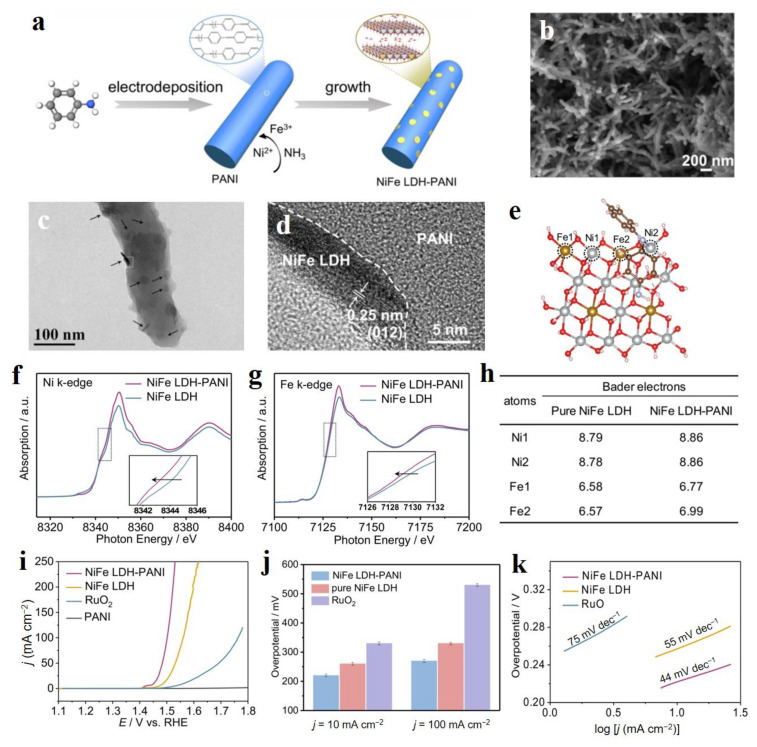
Preparation, characterizations, and OER performance of NiFe-LDH-PANI. (**a**) Schematic illustration of the synthesis of NiFe-LDH-PANI. (**b**) SEM, (**c**,**d**) TEM images of NiFe LDH-PANI. (**e**) The structural model of NiFe-LDH-PANI. Gray, yellow, red, brown, light blue, and pink spheres present Ni, Fe, O, C, N, and H atoms, respectively. (**f**) Ni K-edge and (**g**) Fe K-edge XANES spectra. (**h**) Bader charges for Ni and Fe atoms at the surface of pure NiFe-LDH and the interface of NiFe-LDH-PANI. (**i**) LSV curves of serial catalysts. (**j**) Overpotentials at different current densities. (**k**) Tafel slope. (**a**–**k**) Reproduced with permission [113]. Copyright 2021, Elsevier.

Due to the easier adjustability of interlayer spacing, the interlayer metal ions of LDHs are capable of coordinating with organic ligands, including the conductive polymers and metal-organic framework to construct organic/LDHs heterojunctions to enhance the electrocatalytic activity of LDH [111,112,113,114]. Huang et al. prepared the electron-rich NiFe-LDH loaded on carbon paper modified by polyaniline (PANI) via the first electrodeposition and followed by the hydrothermal method (Figure 13a) [113]. SEM and TEM images showed that NiFe-LDH nanodisks grew on the surface of PANI nanorods with intimate contact (Figure 13b–d). Due to this point, the strong chemical interaction between NiFe-LDH and PANI resulted in the changed electron state of Ni and Fe sites in NiFe-LDH. In detail, Ni and Fe gathered more electrons from PANI, leading to the negative shift of pre-edge for Ni and Fe compared to pure NiFe-LDH (Figure 13f,g) and lower binding energies in their XPS spectra. Bader charge analysis showed that Ni and Fe sites in NiFe-LDH-PANI had a higher electron density compared to pure NiFe-LDH (Figure 13e,h), and higher DOS near the Fermi level. Because of this point, NiFe-LDH-PANI possessed a more moderate energy for the formation of *OH and a lower energy for the formation of *O, thus lowering the energy barrier of the reaction and accelerating the kinetics of OER. Finally, NiFe-LDH-PANI delivered an excellent OER performance including the low overpotentials of 220 and 270 at 10 and 100 mA cm^−2^, respectively (Figure 13i), which is much better than that of commercial RuO_2_ (Figure 13j), as well as a low Tafel slope of 44 mV dec^−1^ (Figure 13k).

In addition, a metal organic framework (MOF) also can form the heterojunction with LDH materials to enhance catalytic performance [112,115,116]. Chen’s group prepared a heterogeneous array of 2D NiFe-LDH nanosheets packed with 1D NiFe MOF (NiFe-LDH/MOF) with an extraordinary OER performance in carbon fiber cloth [115]. During the preparation process, NiFe-LDHs were partially converted into NiFe MOFs via the template sacrifice method. In the formation process of the catalyst, 2,5-dihydroterephtalic acid (H_4_DOBDC) coordinated with NiFe-LDHs, which led to an in situ phase reconstruction and nanocrystalline growth. With the increase in reaction time, the pristine NiFe-LDHs gradually transformed into ultrathin NiFe-LDH nanosheets and Fe/Ni-based MOF. This unique method endowed the intimate contact of NiFe-LDH nanosheets and Fe/Ni-based MOF, thus delivering a superior OER performance, including a low overpotential of 72 mV 100 mA cm^−2^ and high stability in 36 continuous tests and 10,000 potential cycles. In this material system, the unsaturated porous NiFe MOFs provided the abundant metal active sites and facilitated the formation of the ultrathin NiFe-LDHs, which made more OER active sites be exposed in the electrolyte. This work also provides an efficient approach to construct advanced electrocatalysts to decrease the size in thickness of LDH via a template sacrifice method.

## 4. Morphological Strategy

The electrocatalytic performance of catalysts is closely related with their morphologies, which involve the number of active sites, the electron transfer process between bulk catalysts and reactants, as well as reactant and product diffusion. Nowadays, some reported morphological strategies of LDH have been reported to enhance the activity of LDH materials. In this section, the recent advances of morphological strategy on enhancing electrocatalytic performance will be summarized including preparation methods, characterizations, the electrocatalytic performance, and the reaction mechanism.

### 4.1. Intercalation

Due to the weak interaction of layers for LDH, the anions are easily incorporated into layers of LDH, thus tuning their interlayer spacing distance and enhancing their ECSA. In general, the large interlayer spacing facilitates the formation of LDHs nanosheets with high specific surface area and exposed density of active sites, as well as forming anion defects, thus further improving the electrocatalytic performance of LDHs. In addition, when the spacing between layers exceeds a certain threshold, it is helpful for LDHs to peel off and form an ultrathin nanosheet structure with a smaller size.

**Figure 14:**
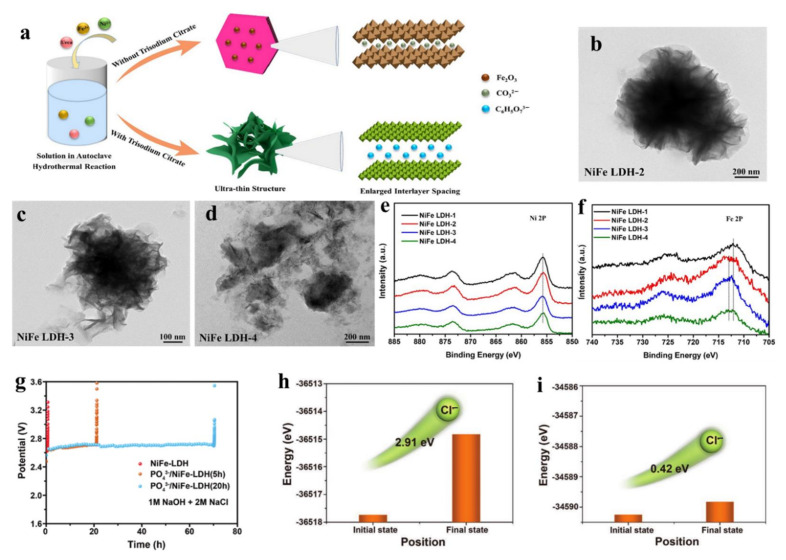
Preparation illustration, characterizations, and theoretical studies of LDH intercalated by anion. (**a**) Schematic illustration of the synthesis process of the NiFe-LDH without and with trisodium citrate. (**b**–**d**) TEM images of (**b**) NiFe LDH-2, (**c**) NiFe LDH-3, and (**d**) NiFe LDH-4. (**e**) Ni 2p and (**f**) Fe 2p XPS spectra of NiFe LDH-1, NiFe LDH-2, NiFe LDH-3, and NiFe LDH-4. (**g**) Durability tests of the NiFe-LDH and the PO_4_^3−^/NiFe-LDH with different intercalation times under the current density of 400 mA cm^–2^. (**h**,**i**) Energy barrier for Cl^−^ migration in the PO_4_^3−^/NiFe-LDH (**h**) and pure NiFe-LDH (**i**). (**a**–**f**) Reproduced with permission [117]. Copyright 2022, Elsevier. (**g**–**i**) Reproduced with permission [118]. Copyright 2022, Wiley-VCH.

Gu et al. realized the preparation of intercalation-induced partial exfoliation of NiFe-LDHs with abundant active edge sites by utilizing the one-pot hydrothermal method [117]. In the preparation process, trisodium citrate was the intercalation agent replacing the CO_3_^2−^ ion intercalation in the traditional NiFe-LDH material, which endowed it being of a large interlayer spacing and formed an obviously different morphological structure (Figure 14a). The morphology of NiFe-LDH changed from the flower-like structure composed of nanosheets to the ultrathin nanosheets structure with the increase of trisodium citrate content, which suggested that more active sites were exposed on the surface of LDH (Figure 14b–d). XPS data showed that the electronic structure of Fe sites on the NiFe-LDH surface changed with a positive shift after introducing the citrate anion, and the Ni sites kept a stable structure without clear changes (Figure 14e,f). Because of the existing above changes after the intercalation of citrate anion, the modified NiFe-LDH exhibited an enhanced OER activity. Additionally, anion intercalation also can improve the stability of LDH. For instance, Sun et al. prepared NiFe-LDH with PO_4_^3−^ intercalation, which efficiently inhibited the corrosion of Ni conductive substrate by Cl^−^ in seawater due to the existing electrostatic repulsion between highly negatively charged Cl^−^ ions and highly negatively charged PO_4_^3−^, and thus significantly improved the stability of NiFe-LDH in seawater electrolyte [118]. PO_4_^3−^ intercalated NiFe-LDH maintained a high current of 400 mA cm^−2^ after a continuous 72 h test in simulated seawater solution with a high NaCl concentration (1M NaOH+2M NaCl), and its stability was 100 times higher than that of pure NiFe-LDH (40 min) (Figure 14g). DFT calculation showed that NiFe-LDH with PO_4_^3−^ intercalation had a higher migration energy barrier of Cl^−^ than that of pure NiFe-LDH, which proved that Cl^−^ had a weaker migration ability in NiFe-LDH with PO_4_^3−^ intercalation (Figure 14h,i). Therefore, the excellent stability of NiFe-LDH intercalated with PO_4_^3−^ could be attributed to the fact that the introduction of PO_4_^3−^ was conducive to reduce the corrosion of Cl^−^ on the foam nickel conductive substrate and the shedding of NiFe-LDH without Cl^−^ corrosion. This work provides a new idea for designing efficient and stable seawater electrolysis catalysts through the anion intercalation of LDH materials. Nowadays, anion intercalation has been developed as an efficient approach to improve electrocatalytic performance of LDH [119,120,121], which leads to LDH with a thin layer structure, more exposed catalytic sites and tuned electronic densities of metals. Because of these advantages, LDH with anion intercalation always exhibits an excellent activity and stability in water splitting. The layer distance between LDH materials can also be easily tuned by the organic small molecules, in which the organic small molecules efficiently weaken the interlayer interaction of LDH materials, thus leading to the enhanced electrocatalytic activity [122,123,124]. Gao’s group reported that CoFe-LDH materials with a large interlayer distance of 0.6 nm were prepared in the ethanol solution using ammonium bicarbonate (NH_4_HCO_3_) as a pH regulator [122]. NH_4_HCO_3_ was decomposed into H_2_O and NH_3_, thus leading to the forming of CoFe-LDH materials with adsorbing ethanol molecules and a large interlayer distance being of 0.6 nm. CoFe-LDH material featured by a large interlayer distance possessed the more cation defects supported by XAS spectra of Co and Fe compared to CoFe-LDH material without ethanol, and the low impedance of electron transfer, as well as a large ECSA, which jointly contribute to the enhanced OER activity. Recently, Sun’s group have prepared a benzoate (BZ) anions-intercalated NiFe-LDH (BZ-NiFe-LDH) nanosheet [123]. Due to the resistance of BZ to Cl^−^ over NiFe-LDH, the BZ-NiFe-LDH nanosheet exhibited a superior OER activity and stability in seawater splitting. In detail, the decreased OER activity for the BZ-NiFe-LDH nanosheet is about 28% in the continuous 100 h test, much lower than that of NiFe-LDH being 79%. This important work provides a promising method to construct advanced OER catalysts with superior stability though the organic molecule intercalation in LDH materials, and extends the application of LDH materials to seawater electrolysis.

### 4.2. Exfoliation

In general, the traditional LDHs are layered stacked structures, which not only shield a large number of catalytic active sites, but also inhibit the electron and mass transfer during electrocatalytic reactions over the catalyst. Therefore, it can be stripped into ultrathin nanosheets with smaller sizes by the exfoliation strategy to significantly enhance electrocatalytic performance [125,126,127,128,129]. The peeled single-layer ultra-thin LDHs nanosheets provide a large surface area and more electrochemically active sites, thus facilitating the reaction with reactants.

**Figure 15:**
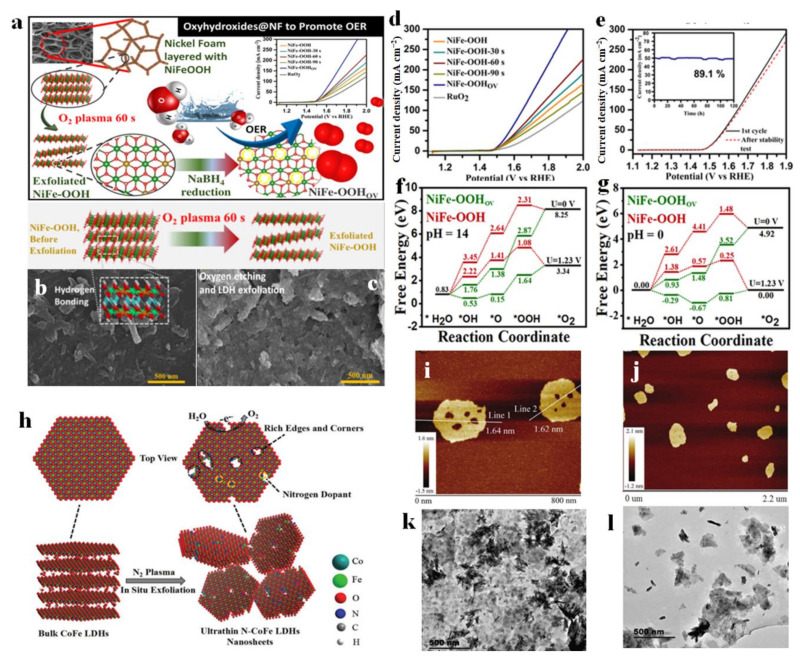
Preparation illustration, characterizations, and theoretical studies of LDH exfoliation. (**a**) Schematic illustration of structural tuning in NiFe-OOH grown on Ni Foam as the substrate to fabricate NiFe-OOH_OV_ catalyst for OER. (**b**,**c**) SEM images of (**b**) NiFe-OOH and (**c**) NiFe-OOH after 60 s of O-plasma exposure (NiFe-OOH 60 s). (**d**) OER polarization curves of NiFe-OOH_OV_, NiFe-OOH-30 s, NiFe-OOH-60 s, NiFe-OOH-90 s, and NiFe-OOH catalysts compared to RuO_2_ in 1.0 M KOH electrolyte. (**e**) OER polarization curves for NiFe-OOH_OV_ before and after long-term stability test. The inset shows the chronoamperometric response at a current density of 50 mA cm^−2^ for 120 h. (**f**,**g**) Free energy diagrams of NiFe-OOH and NiFe-OOH_OV_ for the OER at different pH: (**f**) pH = 0 and (**g**) pH = 14. (**h**) Illustration of the exfoliation of bulk CoFe-LDHs into ultrathin CoFe-LDHs nanosheets by N_2_ plasma. (**i**,**j**) AFM images of the ultrathin N-CoFe LDHs nanosheets. (**k**,**l**) TEM of (**k**) NiFe LDH and (**l**) Exf NiFe-LDH with 15 min. (**a**–**g**) Reproduced with permission [125]. Copyright 2021, American Chemical Society. (**h**–**j**) Reproduced with permission [126]. Copyright 2018, Wiley-VCH. (**k**,**l**) Reproduced with permission [127]. Copyright 2021, Elsevier.

Bagchi et al. stripped NiFe-LDH by analogy with graphene stripping technology and prepared ultra-thin NiFeOOH nanosheets with oxygen vacancies by subsequent reduction treatment [125]. In the synthesis process, layered LDHs were firstly stripped by the oxygen plasma method, and then reduced by NaBH_4_ at room temperature, which not only produced vacancies, but also inhibited the formation of hydrogen bonds connecting the layers together, which was conducive to the stripping of LDH (Figure 15a). Compared with pure NiFe-OOH, NiFe-OOH after treated O-plasma for 60 s (NiFe-OOH 60 s) showed the thinner fragment structure (Figure 15b,c), thus leading to the excellent OER performance. It delivered a current density of 50 mA cm^−2^ with a low overpotential of 330 mV for OER, and also maintained an ultra-high stability of 120 h at this current density (Figure 15d,e). The DFT calculation at different PH values revealed that NiFe-OOH 60 s was prone to adsorb O *, OH *, and OOH * than pristine NiFe-OOH, and the free energy profile of OER steps was optimized due to the existence of oxygen defects (Figure 15f,g). In this material system, the *V*o vacancy facilitated the electron transfer process during reactions, thus leading to the fast electron transfer to participate in reactions. Meanwhile, NiFe-OOH_VO_ material possessed a large ECSA, indicating its more exposed active sites in the electrolyte, which also facilitated the electrocatalytic reactions. Wang et al. prepared vacancy-rich N-doped ultrathin CoFe-LDH nanosheets by N_2_ plasma etching technology, as shown in Figure 15h [126]. The AFM image of the sample showed that the stripped N-CoFe LDH had a thickness of only 1.6 nm (Figure 15i,j). At the same time, many atomic-sized pores were observed on the basal plane of N-CoFe LDH due to plasma treatment, which proved the structural characteristics of the catalyst with rich edge sites, and the atomic-sized pores were helpful to improve the electrocatalytic activity of the catalyst due to the exposure of more electrocatalytic active sites, as presented by a lower resistance (21.4 Ω) than that of CoFe LDH (94.9 Ω). Meanwhile, the formed atomic-sized pores resulted in the generated Co and Fe with low valence states (Co^2+^ and Fe^2+^) which facilitated the adsorption of H_2_O molecules, thus enhancing OER activity. Benefiting from the unique structural characteristics, N-CoFe LDHs/NF showed excellent electrocatalytic activity, and only an ultra-low overpotential of 233mV was needed to achieve the geometric catalytic density of 10 mA cm^−2^. Additionally, the ultrasonic treatment is one of the indispensable methods to peel off 2D layered materials. Zheng et al. explored the effect of exfoliating NiFe-LDH on the performance of the catalyst OER under different ultrasonic conditions (including suspension concentration, sonification times, and amplification in water) [127]. TEM images showed that the stacked NiFe-LDH nanolayers were peeled off into ultra-thin layered structures, and the thickness could even reach 0.227 nm (Figure 15k,l). Because of this point, NiFe-LDH showed excellent OER activity with the overpotential of 250 mV at 10 mA cm^−2^ in alkaline solution. In addition, the ratio of Fe with a +3 valence state in NiFe-LDH with 0.227 nm in thickness was larger than that of bulk NiFe-LDH material, which is widely regarded as highly active catalytic sites for OER, thus facilitating the OER reaction steps. In general, exfoliation is an efficient method to generate the thin NiFe-LDH layer with abundant exposed sites and accompanied by generated defect vacancies to form highly active catalytic sites, thus providing more sites for the reaction and exhibiting high-electrocatalytic performance.

## 5. Vacancy Strategy

Currently, the vacancy strategy is also recognized as an indispensable approach to improve the initial electrocatalytic performance of Ni (Co)-based LDH materials. Generally, the vacancy regulation of LDHs includes cation vacancy (e.g., M^2+^/M^3+^, where M is metal cation) regulation and anion vacancy (e.g., oxygen vacancies) regulation. The generation of oxygen vacancies means the generation of a large number of unsaturated atoms, which can significantly enhance the surface adsorption energy of LDHs for anions (OH^—^) in electrolyte and reduce the activation energy of the full reaction steps. The existence of metal vacancies can provide a large number of unsaturated coordination atoms, which regulate the electronic configuration near the active metal sites, thus promoting HER/OER activity.

**Figure 16:**
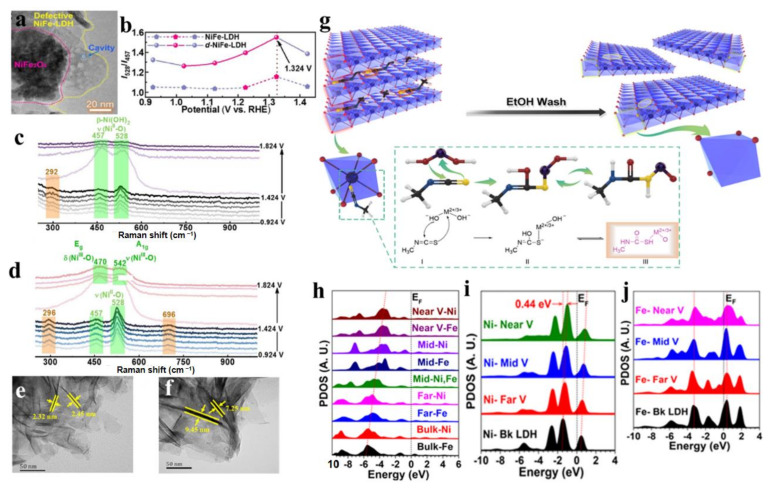
Characterization and DFT results for the vacancy strategy to enhance electrocatalytic performance of LDH materials. (**a**) TEM images of d-NiFe-LDH. (**b**) In situ Raman spectroscopy measurements of the evolution of I_528_/I_457_ versus potential. (**c**) The Raman spectra of pristine NiFe-LDH under different applied potentials. (**d**) The Raman spectra of pristine d-NiFe-LDH under different applied potentials. (**e**,**f**) TEM images of (**e**) NiFe LDH and (**f**) v-NiFe LDH. (**g**) The mechanism of the NiFe-LDH with metal and oxygen multivacancies. (**h**) The site-dependent PDOSs of O-2p orbitals. (**i**) The site-dependent PDOSs of Ni-3d orbitals. (**j**) The site-dependent PDOSs of Fe-3d orbitals. (**a**–**d**) Reproduced with permission [131]. Copyright 2021, Wiley-VCH. (**e**–**j**) Reproduced with permission [132]. Copyright 2020, Elsevier.

Heteroatom doping and plasma treatment are the main methods used for generating vacancies [31,41,125,126,130]. Furthermore, Li et al. reported a defensive NiFe-LDH with metal vacancies (d-NiFe-LDH), which was prepared by dissolving metal cations from NiFe-LDH with polar aprotic solvent [131]. In the synthesis process, part of Ni^2+^ and Fe^3+^ were dissolved in the high-temperature solvent heat treatment of a mixed solution of ethanol, water, and N, N-dimethylformamide, and then the weak alkaline environment generated by DMF hydrolysis made the two cations re-deposit on the remaining LDH in the form of NiFe_2_O_4_. TEM images clearly showed the formation of NiFe_2_O_4_ and some cavities left by cation dissolution (Figure 16a). In-situ Raman spectroscopy revealed that with the increase of applied voltage, I_528_/I_457_ (the intensity ratio of the characteristic peaks at 528 and 457cm^−1^), regarded as the index of the fraction of defective or disordered Ni(OH)_x_ species, increased (Figure 16b–d). the cation vacancy *V*_M_, initially generated in the prepared samples, gradually transformed into the *V*_M_-OH configuration, and then further transformed into the *V*_M_OH-H configuration with the most active thermodynamic properties, which proved that the formation of vacancies promoted the structural transformation of NiFe-LDH during the OER reaction.

In addition, vacancies can also be generated by electron-withdrawing organic small molecules. Yang et al. used small organic molecule methyl isocyanate (CH_3_NCS) to create multiple vacancies of metal and oxygen on the edge and basal surface of LDH, which successfully enhanced the catalytic activity of LDHs for OER [132]. In the process of synthesis, the electron-withdrawing organic small molecule CH_3_-N=C=S entered the LDH interlayer as an anchoring agent, which caused the LDH to peel off (Figure 1g). Then, CH_3_NCS anchored on the specific atoms (oxygen and metal atoms) of the MO_6_ unit in LDH, and removed the oxygen and metal atoms in LDH, thus forming multiple vacancies of oxygen and metal in LDH. TEM showed that the thickness of the modified NiFe-LDH nanosheets was reduced from 8 nm to 2.5 nm, which was helpful for exposing more unsaturated catalytic active sites (Figure 1e,f). At the ultra-low overpotential of 260 mV, the current for an OER of 50 mA cm^−2^ could be delivered, and its performance was better than that of most reported recent state-of-the-art catalysts. DFT revealed that the interaction of metal vacancies and O vacancies in NiFe-LDH promoted electron transfer (Figure 1h–j). Such a composite defect region was not only beneficial to the electrical activity of the intermediate product, but also was beneficial to the stability of the intermediate product. The modulated electronic environment determined the superior OER performance. Based on the above analysis, it is easily concluded that the vacancy strategy is an efficient approach to enhance the electrocatalytic performance of LDH materials, which also has developed as a convenient method to construct superior LDH materials [133,134].

## 6. Summary and Perspective

This review elaborates on the comprehensive achievements of Ni (Co, Fe)-based LDH 2D materials for water splitting, including their preparation methods and strategies, electrocatalytic performances, characterizations, and electrocatalytic mechanisms over catalysts. For chemical modification over Ni (Co, Fe)-based LDH 2D materials, there are mainly three strategies to modify LDH materials towards a high electrocatalytic performance in water splitting. The first one is the heteroatom doping strategy, which can effectively tune the electron distribution of host atoms due to the formed new chemical bonds between heteroatoms and host atoms. Meanwhile, doping with heteroatoms can easily produce a large number of oxygen vacancies (*V*_O_) due to the broken crystal structure, which can increase the conductivity of LDH and also make more active sites be exposed in the electrolyte, thus leading to easier electron transfer during reactions and to an increased number of catalytic sites reacting with intermediates. Additionally, the generated *V*_O_ and heteroatoms jointly regulate the electronic states of catalytic sites, thus optimizing the adsorption energies of intermediates. In general, heteroatom doping contains the precious metal, non-precious metal, and non-metal doping three categories, and these heteroatoms lead to the generation of new highly active catalytic sites for water splitting.

The second one is the heterojunction strategy which can generate new highly active catalytic sites in the interface between two compounds due to their strong chemical interaction. Such a strong chemical interaction also tunes the electronic state of individual compounds compared to pure compounds. In general, there exist two functions of heterojunction for realizing the high electrocatalytic performance of LDH-based catalysts. Firstly, the enhanced conductivity of LDH-based materials can be obtained through hybridizing with high conductive metal compounds, which efficiently improve the electron transfer rate between reactants and catalysts, thus greatly facilitating the reaction dynamics. Secondly, new catalytic sites are formed between the two compounds, which can optimize the free energy profiles of reactions, thus lowering the overpotentials. Nowadays, the heterojunction strategy for constructing LDH-based catalysts hybridized by other compounds includes the metals, metal compounds, carbon, and organic materials, which can also exist as bifunctional catalytic sites for HER and OER. The third one is the morphology strategy to regulate the size of LDH in thickness, which can efficiently increase the number of active sites, enhance the electron transfer process between bulk catalysts and reactants, as well as facilitate reactant and product diffusion, and thus greatly enhance the electrocatalytic performance of water splitting.

**Figure 17:**
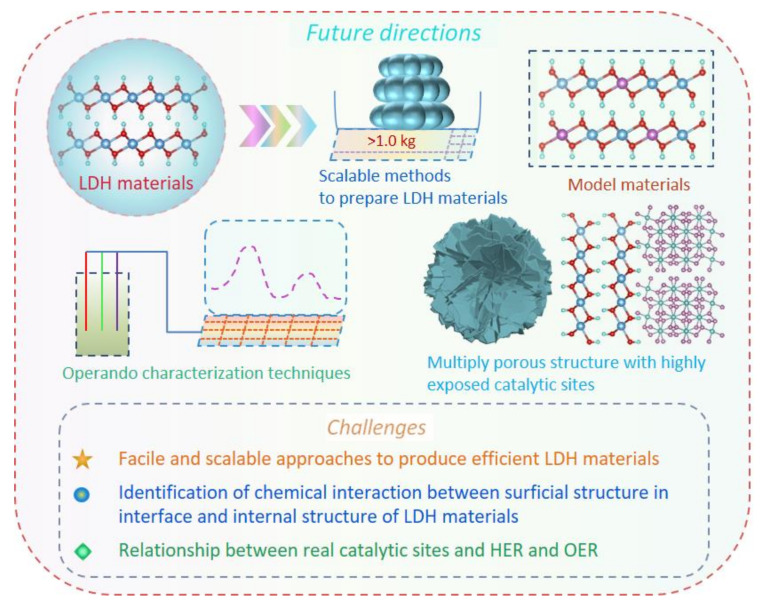
Major challenges and future directions in the design of advanced LDH materials for water splitting.

Nowadays, with the development of characterization instruments, the microstructures of LDH, as well as evolution of chemical environmental changes during reactions, have been solidly established by combining multiply characterization technologies. Simultaneously, the reaction mechanism over electrocatalysts can also be reasonably proposed, especially for the intermediate detection, e.g., OOH*, OH*, and H* species. Despite the rapid progress in water splitting for LDH materials, several major challenges still need to be addressed for the construction of advanced LDH materials to enable their application in water splitting (Figure 17). The current critical issues and possible future directions in the water splitting field of LDH are highlighted in the following parts.

1. The practicality of convenient and scalable approaches is a prerequisite for the implementation of high-performance LDH materials with precisely tuned electronic structures for water splitting. The target of substituting precious-metal based electrocatalysts with abundant transition metals should be achieved through the convenient and scalable synthetic strategies to satisfy the large market demands of water splitting technology to obtain hydrogen energy. Despite the promising electrocatalytic performance compared to commercial precious metal-based materials, Ni (Co, Fe)-based LDH materials have not been widely applied in industrial water splitting technology, presumably due to the difficulty in the large-scalable synthetizing of LDH and does not satisfy the demand of the large-scale application of water splitting.
2. The chemical interaction between the surficial structure in interface and internal structure of LDH materials is critical for the design and construction of LDH materials to realize excellent electrocatalytic performance, including activity and stability. Although the chemical states of metal atoms in LDH materials can be clearly observed during reactions, the amount of new formed metal sites can be not quantized, as well as the difficulty in revealing the detailed information in the interface between individual components. Therefore, the newly formed species in the interface should be considered carefully in tailoring electrocatalytic activity, which involves the formation of highly active catalytic sites and the electronic changes of LDH.
3. Model catalysts with precisely stable microstructures are explored to investigate the reaction mechanism, which provides the reaction rate limiting step of HER and OER over catalysts. Guided by this principle, the Ni (Co, Fe)-based LDH can be easily constructed, even in design bifunctional HER and OER materials. In many cases, the local structure of LDH is an average result due to the difficulty in distinguishing the ratios of each structure, e.g., OH* or O* bonded with the metal. Therefore, it is highly desirable to develop model catalysts to probe the structural evolution of LDH during water splitting reactions through in situ/operando characterization techniques.
4. Multiplying porous structures with highly exposed single atoms is preferred to realize the metals to participate in the reaction, the fast reactant transport, and efficient gas product dissociation. HER and OER are the three-phase reactions referring to the gas, liquid, and solid surface of catalysts. The porous structure with highly exposed single metal atoms is conducive to the reactant chemical acting with the catalytic site as soon as possible, meanwhile facilitating gas product dissociation and reactant transport, thus endowing a high efficiency of H_2_ production from water splitting, presenting a high current density. The generated strain in the porous structure likely tailors the chemical environments of LDH, thus regulating its catalytic performance.

Ni (Co, Fe)-based LDH materials in water splitting have achieved much progress, including their preparation methods, catalytic mechanism, and electrocatalytic performance. In this review, we categorize the three strategies to modify LDH according to their formation and regulation mechanisms, which involves their preparation methods, microstructure, catalytic mechanism, and electrocatalytic performance. The joint effort between experimental and theoretical groups will offer new viewpoints in principle to develop highly efficient LDH in water splitting. The increased intensive research will help to address the critical challenges and thus accelerate the development of new LDH with high activity and stability in water splitting.

## Data Availability

Data are contained within the article.
